# GPR39 Deficiency Impairs Memory and Alters Oxylipins and Inflammatory Cytokines Without Affecting Cerebral Blood Flow in a High-Fat Diet Mouse Model of Cognitive Impairment

**DOI:** 10.3389/fncel.2022.893030

**Published:** 2022-07-06

**Authors:** Thierno M. Bah, Elyse M. Allen, Manuel Garcia-Jaramillo, Ruby Perez, Yalda Zarnegarnia, Catherine M. Davis, Madeline B. Bloom, Armando A. Magana, Jaewoo Choi, Gerd Bobe, Martin M. Pike, Jacob Raber, Claudia S. Maier, Nabil J. Alkayed

**Affiliations:** ^1^Department of Anesthesiology and Perioperative Medicine, Oregon Health & Science University, Portland, OR, United States; ^2^Department of Chemistry, Oregon State University, Corvallis, OR, United States; ^3^Department of Environmental and Molecular Toxicology, Oregon State University, Corvallis, OR, United States; ^4^Department of Behavioral Neuroscience, Oregon Health & Science University, Portland, OR, United States; ^5^Linus Pauling Institute, Oregon State University, Corvallis, OR, United States; ^6^Advanced Imaging Research Center, Oregon Health & Science University, Portland, OR, United States; ^7^Departments of Neurology, Radiation Medicine, and Psychiatry, Division of Neuroscience, Oregon National Primate Research Center, Oregon Health & Science University, Portland, OR, United States; ^8^College of Pharmacy, Oregon State University, Corvallis, OR, United States; ^9^Knight Cardiovascular Institute, Oregon Health & Science University, Portland, OR, United States

**Keywords:** GPR39, vascular cognitive impairment, dementia, oxylipins, cerebral blood flow, high fat diet, neuroinflammation, mouse

## Abstract

Vascular cognitive impairment (VCI) is the second most common cause of dementia. There is no treatment for VCI, in part due to a lack of understanding of the underlying mechanisms. The G-protein coupled receptor 39 (GPR39) is regulated by arachidonic acid (AA)-derived oxylipins that have been implicated in VCI. Furthermore, GPR39 is increased in microglia of post mortem human brains with VCI. Carriers of homozygous GPR39 SNPs have a higher burden of white matter hyperintensity, an MRI marker of VCI. We tested the hypothesis that GPR39 plays a protective role against high-fat diet (HFD)-induced cognitive impairment, in part mediated via oxylipins actions on cerebral blood flow (CBF) and neuroinflammation. Homozygous (KO) and heterozygous (Het) GPR39 knockout mice and wild-type (WT) littermates with and without HFD for 8 months were tested for cognitive performance using the novel object recognition (NOR) and the Morris water maze (MWM) tests, followed by CBF measurements using MRI. Brain tissue and plasma oxylipins were quantified with high-performance liquid chromatography coupled to mass spectrometry. Cytokines and chemokines were measured using a multiplex assay. KO mice, regardless of diet, swam further away from platform location in the MWM compared to WT and Het mice. In the NOR test, there were no effects of genotype or diet. Brain and plasma AA-derived oxylipins formed by 11- and 15-lipoxygenase (LOX), cyclooxygenase (COX) and non-enzymatically were increased by HFD and GPR39 deletion. Interleukin-10 (IL-10) was lower in KO mice on HFD than standard diet (STD), whereas IL-4, interferon γ-induced protein-10 (IP-10) and monocyte chemotactic protein-3 (MCP-3) were altered by diet in both WT and KO, but were not affected by genotype. Resting CBF was reduced in WT and KO mice on HFD, with no change in vasoreactivity. The deletion of GPR39 did not change CBF compared to WT mice on either STD or HFD. We conclude that GPR39 plays a role in spatial memory retention and protects against HFD-induced cognitive impairment in part by modulating inflammation and AA-derived oxylipins. The results indicate that GPR39 and oxylipin pathways play a role and may serve as therapeutic targets in VCI.

## Introduction

The number of individuals with dementia, estimated at 57.4 million worldwide, is anticipated to triple by 2050 at a cost approaching $4 trillion (GBD 2019 Dementia Forecasting Collaborators, 2022). VCI is the second most common cause of dementia after AD (Iadecola et al., 2019). The prevalence of VCI increases with age and is estimated to affect more than 30% of people over 80 years of age (Gorelick et al., 2011). There is currently no disease-modifying therapy that can prevent or treat VCI, in part due to lack of understanding of underlying mechanisms (Pantoni, 2010; O’Brien and Thomas, 2015). The most common cause of VCI is cerebral small vessel disease (SVD). The causes of SVD-related dementia are not completely understood, but they include a combination of genetic, environmental and lifestyle factors. Among the modifiable risk factors that have been linked to increased risk of dementia in late-life is mid-life metabolic syndrome (Whitmer et al., 2005). Chronic HFD in mice is associated with similar vascular, metabolic, and cognitive changes as in human (Whitmer et al., 2005).

Among the potential mediators of SVD are oxylipins, a collection of bioactive lipid metabolites that play important roles in brain health and disease, including small vessel functions and disorders. Oxylipins are formed by enzymatic and non-enzymatic oxidation of polyunsaturated fatty acids (PUFAs) including AA, EPA, DHA, LA, and ALA (Gabbs et al., 2015). The three main enzymatic pathways involved in oxylipins production are the COX, LOX, and cytochrome P450 enzymes (CYP). COX-derived prostaglandins are generally considered pro-inflammatory. LOX-derived mid-chain alcohols, leukotrienes, and ketones can be either pro- or anti-inflammatory (depending on the LOX isoform, the parent fatty acid and hydroxylation position). CYP-derived epoxides are considered anti-inflammatory, while CYP-derived hydroxyeicosanoids are considered pro-inflammatory (Davis et al., 2017; Liu et al., 2018). Metabolic syndrome resulting from chronic HFD in mice is associated with a disruption of oxylipins pattern and a switch to a mostly proinflammatory oxylipins profiles (Shearer et al., 2018).

We recently reported that the G protein-coupled receptor 39 (GPR39) is a dual-ligand receptor regulated by AA-derived oxylipins with opposing actions on inflammation and microvascular tone (Xu et al., 2021; Alkayed et al., 2022). GPR39 is a member of the ghrelin family of G protein-coupled receptors (GPCRs) that has recently been implicated in VCI (Davis et al., 2021a). Furthermore, we also observed increased GPR39 expression in microglia in *post mortem* aged human brains (Davis et al., 2021a). Importantly, carriers of homozygous GPR39 single nucleotide polymorphisms (SNPs) have higher white matter hyperintensity burden, a MRI marker for VCI (Davis et al., 2021a). However, it is not clear if the association between GPR39 expression and SNPs are causally and mechanistically linked to the development of VCI. Therefore, in this study, we used GPR39 mutant mice to determine if GPR39 deficiency affects cognition at baseline and alters HFD-induced cognitive impairment. We tested the hypothesis that GPR39 plays a protective role against cognitive impairment by modulating CBF and neuroinflammation. We also measured brain oxylipins profiles in brains of GPR39 KO and WT mice to determine if HFD and GPR39 deletion induce a pro-inflammatory oxylipins signature as a possible contributor to the changes in CBF, neuroinflammation and cognition.

## Materials and Methods

Supplementary Material detailing the experimental methods and results accompanies this paper.

### Animals and Study Design

All procedures involving the use of animals were approved by the Oregon Health & Science University (OHSU) Institutional Animal Care and Use Committee (IACUC) and in compliance with the U.S. Department of Agriculture Animal Welfare Act and the National Institutes of Health policy on Humane Care and Use of Laboratory Animals.

Thirty-six 4-month-old male mice of one of three genotypes: homozygous GPR39 KO, heterozygous (Het), or WT littermates were used in this study. The GPR39 mutant mice were generated by Cyagen Biosciences (Santa Clara, CA, United States) using a CRISPR/Cas9 deletion strategy and zygotic injections, which have previously been reported (Alkayed et al., 2022).

**FIGURE 1:**
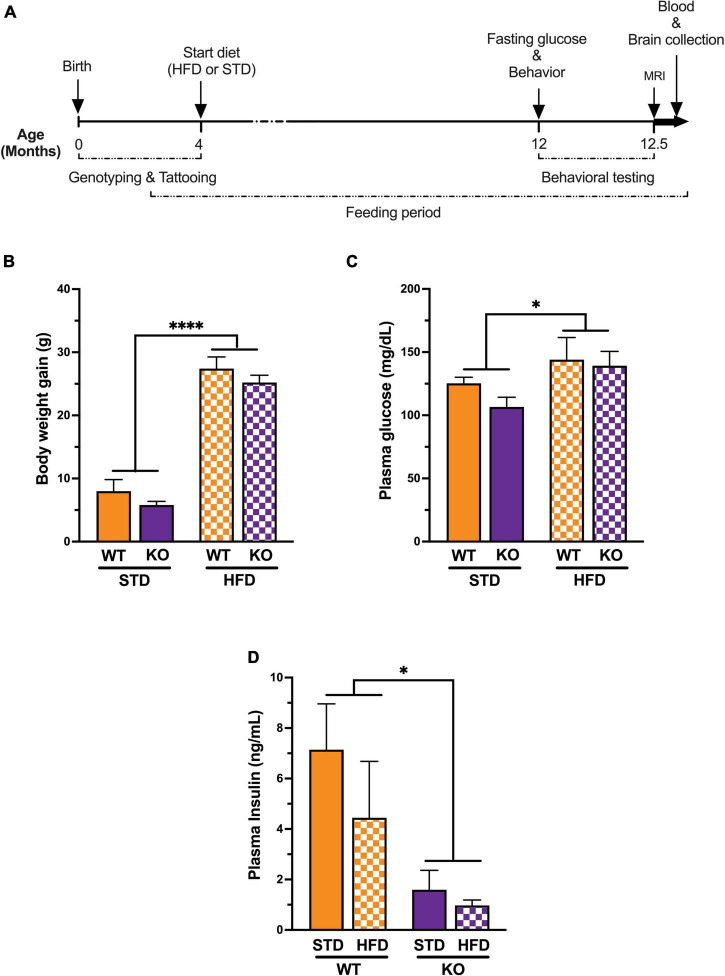
Experimental timeline and Impact of high fat diet on metabolic syndrome. **(A)** All genotypes of mice were placed on a high fat diet or standard diet at 3–5 months of age. Behavioral and cognitive performance was assessed using the water maze and the novel object recognition tests. Fasting glucose was measured just before behavior and magnetic resonance imaging (*n* = 6 per group, randomly selected) upon completion of the study. Plasma samples and brains were collected for biochemical assays and oxylipins analysis respectively. **(B)** Mice on a long-term high fat diet develop a diabetic phenotype. Prior to initiation of dietary intervention, there were no significant difference in body weight between groups. High fat diet caused a significant increase in body weight in both genotype after 8 months on the diet. **(C)** Plasma fasting glucose showed significant diet effect without genotype effect. **(D)** Plasma insulin levels showed significant genotype effect with low insulin in KO mice compared to WT littermates regardless of diet. All values represent means ± standard error of the mean, *p < 0.05, ^****^p < 0.0001, *n* = 5–10 per group.

The animals were group-housed in an animal room colony (5 per cage) under a 12/12-h light/dark cycle (light onset at 06:00 = zeitgeber time [ZT] 0) at 21–23°C ambient temperature and 30–42% relative humidity. An overview of the experimental timeline is presented in Figure 1A. At 4 months of age, the animals were randomly assigned to either high-fat diet (HFD: 60 kcal% fat; D12492i, Research Diets, New Brunswick, NJ, United States) or standard (STD) chow (13 kcal% fat, PicoLab Laboratory Rodent Diet, 5LOD, LabDiet, St. Louis, MO, United States), with food and water available *ad libitum* throughout the study. After 8 months on the diets, cognitive performance was evaluated using the Novel Object Recognition (NOR) and the Morris water maze tests, followed by CBF measurement with and without hypercapnia, to assess vasoreactivity, using ASL MRI. Upon completion of the study, blood was collected by cardiac puncture, and mice were transcardially perfused with heparinized cold saline (1 unit/mL) to remove blood from circulation. Brain tissues were then removed and flash frozen for oxylipins analysis. Collected plasma samples were stored at −80°C for subsequent measurements of insulin and cytokines/chemokines.

Behavioral testing and MRI were completed by experimenters blinded to the experimental and treatment groups. Due to differences in food color and mouse appearance, HFD status could not be blinded during behavioral testing or MRI. All data collected were analyzed by investigators blinded to both diet and treatment status. Reporting of results conforms to the ARRIVE 2.0 (Animal Research Reporting in *In Vivo* Experiments) guidelines.

### Behavioral and Cognitive Testing

Mice were behaviorally tested over 14 days. Mice were tested for exploratory activity and measures of anxiety in the elevated zero maze on day 1 and the open field on days 2 and 3 (Supplementary Materials). Mice were then tested for novel object recognition on days 4 and 5, and for spatial learning and memory in the water maze on days 8 through 12. Finally, mice were tested for contextual and cued fear conditioning on days 13 and 14 (Supplementary Materials). Novel object recognition and water maze tests were performed as described below in detail.

#### Novel Object Recognition

Mice were habituated to an open field chamber over 2 days, with one 5-min trial per day. On day 3, the mice were exposed to the arena containing two identical objects for 15 min. On day 4, one of the objects (henceforth “familiar”) was replaced by a novel object and mice were allowed to explore for 15 min. Performance of the mice was video recorded. Orientation to the object, within 2 cm proximity, as well as interaction with the object (sniffing and pushing) was defined as exploring the object. Videos were analyzed by a researcher blinded to the genotype and diet of the mice. Novel object recognition was calculated as the percent time spent exploring the novel object out of the total time spent exploring both objects. Distance moved was analyzed using automated multiple body point video, using parameters previously described and validated (Benice and Raber, 2008).

#### Water Maze

Hippocampus-dependent spatial learning and memory were assessed in the water maze. The maze consisted of a circular pool (diameter 140 cm), filled with opaque water (24°C), divided conceptually into four quadrants. Mice were first trained to locate an “escape” platform (plexiglass circle, 6 cm radius) submerged 2 cm below the surface of the water and made visible by the use of a cue (a colored cylinder, 2.5 cm radius, 8 cm height) during the “visible” trials (days 1 and 2). For the visible platform training days, there were two daily sessions, morning, and afternoon, which were separated by an intersession interval of 3 h. Each session consisted of two trials, with 10-min inter-trial intervals. Mice were placed into the water facing the edge of the pool in one of nine randomized locations (consistent for each mouse). A trial ended when the mouse located the platform. Mice that failed to locate the platform within 60 s were led to the platform by placing a finger in front of their swim path. Mice were taken out of the pool after they remained on the platform for a minimum of 3 s. During the visible platform sessions, the location of the platform was moved between each of the four quadrants to avoid procedural biases in task learning. After the visual trials, mice were trained to locate a hidden platform, requiring the mice to rely on extra maze cues for spatial reference and orientation. The platform was not rotated during the hidden platform trials and remained in the same location. The 24 h after the last hidden platform training, spatial memory retention of the mice was assessed in a “probe” trial (no platform) that lasted for 60 s. During the probe trial, mice were placed into the water in the quadrant opposite of the target quadrant. The time spent in the target quadrant compared to the time spent in the three non-target quadrants was analyzed. The swimming patterns of the mice were recorded with Noldus Ethovision video tracking software (Ethovision XT, Noldus Information Technology, Wageningen, Netherlands) set at six samples per second. The time to locate the platform (latency), cumulative distance to the platform location, and distance moved were used as a measure of performance for the visible and hidden platform sessions. Because swim speeds can influence the time it takes to reach the platform, those were also analyzed.

### Arterial Spin Labeling Magnetic Resonance Imaging

Magnetic resonance imaging data acquisition and analysis: Imaging was conducted following behavioral testing. MRI was performed at the OHSU Advanced Imaging Research Center using a Bruker-Biospin 11.75 T small animal MR system with a ParaVision 5.1 software platform, 10 cm inner diameter gradient set with a 72 mm (ID) and 60 mm (length) RF resonator for transmitting and an actively decoupled mouse head surface coil for receiving. Mice were anesthetized with a ketamine/xylazine mixture (1.0 mg xylazine/7 mg ketamine/100 g) in combination with low isoflurane (0.75%) in 100% oxygen. The mice were positioned with heads immobilized on an animal cradle. Body temperature of the mice was monitored and maintained at 37°C while monitoring respiration (SA Instruments, Stony Brook, NY, United States). For each mouse, a coronal 25-slice T2-weighted image was acquired (ParaVision spin echo RARE, 256 × 256 matrix, 125 μm in-plane resolution, 0.5 mm slice width, TR 4000 ms, TE effective 23.64 ms, RARE factor 8, 2 averages). These T2-weighted anatomical scans were used for positioning the perfusion image slice at a consistent position approximately 1.75 mm anterior to the anterior commissure. Cerebral blood flow (ml/min/100 g) was measured using ASL, employing the flow-sensitive alternating inversion recovery rapid acquisition with relaxation enhancement pulse sequence (ParaVision FAIR-RARE), with TE/TR = 45.2/10000 ms, slice thickness = 2 mm, number of slices = 1, matrix = 128 × 128, 250 μm in-plane resolution RARE factor = 72, and 23 turbo inversion recovery values ranging from 40 to 4400 ms, and acquisition time of 15 min. This sequence labels the inflowing blood by global inversion of the equilibrium magnetization (Kim, 1995). The ASL sequence was implemented first at the baseline condition with 100% oxygen, and subsequently 10 min after switching to the hypercapnia condition with 95/5% oxygen/carbon dioxide to assess vasoreactivity (or cerebrovascular reactivity), which is a hemodynamic parameter representing the increase in normal cerebral artery blood flow in response to a vasodilatory stimulus such as hypercapnia. Resulting images were analyzed using the Bruker ParaVision software and JIM software (Xinapse Systems LTD, Northants, United Kingdom). Cerebral blood flow maps (ml/100 g-min) were generated using the Bruker ASL perfusion processing macro and exported into JIM for further processing. Outlier value brain pixels (outside 2 SDs) representing large arteries with high, pulsatile flow were excluded, thus arriving at flows which consistently represent tissue microvascular flow. The identical FOV geometry offsets of the T2 and ASL images enabled the ROIs drawn on the T2 image to be readily overlaid onto the corresponding perfusion map for quantification. Mean cerebral blood flow was quantified for the whole brain and for each brain hemisphere, each defined anatomically using the corresponding T2 images.

### Biochemical Assays

Following the acquisition of the MRI data, blood was collected through cardiac puncture at the time of euthanasia. Blood was centrifuged at 2,000 rpm for 15 min to separate the plasma, which was stored at −80°C until assay.

#### Measurement of Plasma Insulin and Multiplex Analysis

Insulin was measured using the ALPCO Ultrasensitive Mouse Insulin ELISA kit (26-G Keewaydin Drive, Salem, NH, Cat # 80-INSMSU-E01, E10) according to manufacturer’s instructions, using the 5 μL protocol. The assay was read at 450 nM on a SpectraMax iD3 multi-label plate reader (Molecular Devices). Mouse cytokines/chemokines were measured using a 26-plex ProcartaPlex Panel 1 antibody-conjugated bead capture assay (Thermo Fisher, EPXR260-26088-901) which enables the exploration of immune function by analyzing 26 protein targets (Supplementary Table 1) in a single well using Luminex xMAP technology. All samples were analyzed on the same plate in duplicate according to the manufacturer’s recommendations. Data was acquired on a Luminex 200 instrument, utilizing a lower bound of 100 beads per sample per analyte. Sample concentrations were calculated from the standard curve using a Logistic 5P regression formula.

#### Oxylipins Measurements

##### Sample Preparation for Oxylipins Analysis

Oxylipins were extracted from brain using the approach previously described (Garcia-Jaramillo et al., 2019), with minor modifications for adaptation to brain tissue. Briefly, brain tissue (∼20 mg) was transferred 2 mL pre-weighted polypropylene tubes containing ceramic beads. Cold LC–MS-grade methanol (35 μL) and an anti-oxidant solution [0.2 mg mL^–1^ solution BHT (butylated hydroxytoluene) in 1:1 methanol: water] (5 μL) was added to each sample. A deuterated oxylipin recovery standard solution (10 μl) was added to each sample. The 10 mM ammonium formate + 1% formic acid in isopropanol (550 μL) and water (100 μL) was added and the tubes were placed in a Precellys^®^ 24 bead-based homogenizer for three cycles of 1 min at 1350 rpm, each. Samples were centrifuged (9000 rpm for 5 min) at 4°C. Supernatants were transferred to a 96-well Ostro Pass Through Sample Preparation Plate (Waters Corporation, Milford, MA, United States) and eluted into glass inserts containing 10 μL 10% glycerol in methanol by applying a vacuum (15 mm Hg) for 10 min. Eluents were dried by vacuum centrifugation in a SpeedVac™ vacuum concentrator for 2 h at room temperature. Once dry, samples were reconstituted with 100 μL of methanol: acetonitrile (50:50), containing the internal standard (CUDA at 50 ng/mL). Samples were transferred to a spin filter (0.22 μm PVDF membrane, Millipore-Sigma, Burlington, MA, United States) and centrifuged (3 min at 9000 rpm) at 4°C before being transferred to 2 mL amber LC–MS vials. Oxylipins from plasma were extracted as previously described (La Frano et al., 2017; Garcia-Jaramillo et al., 2019; Le et al., 2021). Extracts from brain and plasma were stored at −20°C for less than 24 h until analysis by ultra-performance liquid chromatography tandem mass spectrometry (UPLC–MS/MS). The internal oxylipin standards added to the samples were used to correct the recovery of the quantified oxylipins (La Frano et al., 2017; Garcia-Jaramillo et al., 2019; Le et al., 2021).

##### Targeted Oxylipidomics

High performance liquid chromatography (HPLC) was performed using a Shimadzu system (Shimadzu, Columbia, MD, United States) coupled to a QTRAP 4000 (AB SCIEX, Framingham, MA, United States) employing dynamic multi-reaction monitoring (dMRM), as previously described (Garcia-Jaramillo et al., 2019; Le et al., 2021). Briefly, compounds were separated using a Waters Acquity UPLC CSH C18 column (100 mm length × 2.1 mm internal diameter; 1.7 μm particle size) with an additional Waters Acquity VanGuard CSH C18 pre-column (5 mm length × 2.1 mm internal diameter; 1.7 μm particle size) held constant at 60°C. The mobile phase and gradient elution conditions were adopted from Pedersen and Newman (2018). In summary, the mobile phase consisted of (A) water (0.1% acetic acid) and (B) acetonitrile/isopropanol (ACN/IPA) (90/10, v/v) (0.1% acetic acid). Gradient elution conditions were carried out for 22 min at a flow rate of 0.15 mL min^–1^. Gradient conditions were: 0–1.0 min, 0.1–25% B; 1.0–2.5 min, 25–40% B; 2.5–4.5 min, 40–42% B; 4.5–10.5 min, 42–50% B; 10.5–12.5 min, 50–65% B; 12.5–14 min, 65–75% B; 14–14.5 min, 75–85% B; 14.5–20 min, 85–95%B; 20–20.5 min, 95–95% B; 20.5–22 min, 95–25% B. A 5 μL aliquot of each sample was injected onto the column. Oxylipin data obtained by HPLC-dMRM-based analyses was processed using an in-house library embedded in MultiQuant™ software.

### Statistical Analysis

Unless indicated otherwise, data were analyzed by analysis of variance (ANOVA) with Tukey’s and Dunnett’s multiple comparison *post-hoc* tests using diet as the between-subject factor. G*Power 3 (La Frano et al., 2017) was used to calculate the power analysis. As we *a priori* expected a genotype-dependent response to diet, we analyzed the effect of diet in each genotype separately. The number of mice needed to detect an effect of diet for each of the three genotypes based on our previous studies (Johnson et al., 2016; Zuloaga et al., 2016; Davis et al., 2021b) was 12 mice per genotype per diet. All data are shown as mean ± standard error of the mean (SEM) and were first assessed for normality of variance. Statistical analyses were performed using SPSS v.27.0 (IBM Corp., Armonk, NY, United States) and Prism v.9.2.0 (GraphPad Software Inc., San Diego, CA, United States) software. If a violation of sphericity occurred indicating that the variances of the differences between all combinations of the groups were not equal, Greenhouse-Geisser corrections were used. All figures were generated using GraphPad Prism software and we considered *p* < 0.05 as statistically significant.

Linear regression analysis was used to compare the treatment groups in terms of oxylipins values investigating for which oxylipins there was significant difference between treatments. Log transformation of outcome values was performed to improve the model fit in terms of model assumptions and few outliers were excluded from statistical analyses. For Pathway data, for each class of fatty acids, the oxylipins values were summed up based on common pathway first and then linear regression model was fitted to compare the treatment groups for each pathway. *Post-hoc* analysis was performed to compare some groups of interest (e.g., WT vs. GPR39 KO mice) using the Benjamini-Hochberg false discovery rate correction method for multiple comparisons *t*-tests. The results (coefficients, estimates, standard errors, and corresponding p-values, and fold changes) are provided in Tables 1–4. Analyses were conducted using R 4.1.0 (R core Team, Vienna, Austria) (R Core Team, 2021).

**TABLE 1:** Brain oxylipins profile in GPR39 KO and WT littermate mice fed with standard diet and high fat diet.

Class	Oxylipins	Genotype/diet groups	Estimate	SE	*p*-Value	Mean first group	Mean second group	Fold change
**WT vs. KO on HFD (diet + genotype effect)**								
ARA	11(S)-HETE	WT/HFD - KO/HFD	0.942	0.244	0.002	2.368	1.426	2.715
	15(S)-HETE		0.417	0.143	0.021	2.436	2.019	1.512
	PGE2		1.357	0.232	0.000	2.204	0.847	4.371
	PGD2		1.176	0.256	0.000	4.273	3.097	3.450
	PGJ2		0.890	0.208	0.001	1.531	0.641	2.559
	6-keto PGF1a		0.651	0.196	0.008	1.452	0.801	1.961
	8-iso PGF2a		0.569	0.221	0.044	2.844	2.274	1.764
	11 b-PGF2a		0.705	0.256	0.030	2.978	2.273	2.014
	15-keto PGF2a		1.162	0.253	0.000	3.790	2.629	3.397
	13,14-dihydro-15-keto PGE2		0.093	0.028	0.009	−0.309	−0.402	1.097
	13,14-dihydro-15-keto PGD2		0.555	0.165	0.007	0.036	−0.519	1.800
	15-deoxy-delta12,14-PGJ2		0.499	0.132	0.004	−0.143	−0.642	1.674
	Thromboxane B2		0.396	0.137	0.021	2.703	2.307	1.483
	14,15-DiHET		0.941	0.304	0.013	0.517	−0.424	2.606
DHA	7,8-DiHDPA		0.180	0.036	0.000	0.199	0.019	1.199
**WT on STD vs. HFD (diet effect)**								
ARA	11(S)-HETE	WT/STD - WT/HFD	−1.322	0.255	0.000	1.045	2.368	0.243
	15(S)-HETE		−0.559	0.148	0.006	1.877	2.436	0.572
	PGE2		−1.542	0.243	0.000	0.662	2.204	0.185
	PGD2		−1.526	0.267	0.000	2.747	4.273	0.200
	PGJ2		−1.021	0.217	0.001	0.510	1.531	0.331
	6-keto PGF1a		−0.688	0.205	0.008	0.764	1.452	0.484
	8-iso PGF2a		−0.735	0.228	0.021	2.108	2.844	0.481
	11 b-PGF2a		−0.782	0.265	0.030	2.196	2.978	0.457
	15-keto PGF2a		−1.505	0.265	0.000	2.285	3.790	0.204
	13,14-dihydro-15-keto PGE2		−0.108	0.029	0.007	−0.418	−0.309	0.897
	13,14-dihydro-15-keto PGD2		−0.644	0.172	0.006	−0.608	0.036	0.514
	15-deoxy-delta12,14-PGJ2		−0.495	0.138	0.004	−0.638	−0.143	0.601
	Thromboxane B2		−0.507	0.141	0.009	2.196	2.703	0.600
	14,15-DiHET		−1.266	0.317	0.003	−0.749	0.517	0.261
DHA	7,8-DiHDPA		−0.164	0.036	0.000	0.035	0.199	0.848
**WT vs. KO on STD (genotype effect)**								
ARA	PGE2	WT/STD - KO/STD	−0.468	0.214	0.050	0.662	1.130	0.616
	PGD2		−0.545	0.235	0.037	2.747	3.292	0.567
	15-keto PGF2a		−0.532	0.233	0.040	2.285	2.817	0.574
DHA	Resolvin D1		−0.122	0.039	0.023	−0.215	−0.093	0.887

*Brain oxylipins are grouped according to main biosynthetic pathways by (1) FA precursors (i.e., LA, ARA, EPA, and DHA), oxylipin groups (i.e., midchain HODE, EET, mid-chain HETE, EpDPA, DiHDPA), (2) enzymes involved in their synthesis [i.e., oxygenation of PUFAs by LOX followed by reduction or alternatively hydroxylation of PUFAs by CYP1B1; oxidation of PUFAs by CYP450 followed by hydroxylation of oxidized PUFAs by soluble epoxide hydrolase (sEH)], and (3) based on enzymatic product to substrate ratio (i.e., hydroxylation of 10,11-EpDPA to 10,11-DiHDPA, 14,15-EET to 14,15-DiHET, or 19,20-EpDPA to 19,20-DiHDPA by sEH). AA, arachidonic acid; DHA, docosahexaenoic acid; LOX, lipoxygenase; PGD2, prostaglandin D2; PGE2, prostaglandin E2; PGJ2, prostaglandin J2; 11-HETE, 11-hydroxyeicosatrienoic; 15-HETE, 15-hydroxyeicosatrienoic acid; 20-HETE, 20-hydroxyeicosatrienoic acid; 14,15-DiHET, 14,15-dihydroxyeicosatrienoic acid. p-values: are the two-sided p-values associated with the observed statistic (based on t-test form fitting linear regression model), adjusted based on Benjamini–Hochberg false discovery rate correction method for multiple hypothesis testing. Estimate: is the estimated value of the regression term (estimate of coefficient of difference between two groups), SE: The standard error of the regression term (estimate of coefficient of difference between two groups), and fold change: ratio of one group to another group based on the marginal means.*

**TABLE 2:** Brain oxylipins pathways in GPR39 KO and WT littermate mice fed with standard diet and high fat diet.

Class	Oxylipins	Pathways	Genotype/diet groups	Estimate	SE	*p*-Value	Mean first group	Mean second group	Fold change
**WT vs. KO on HFD (diet + genotype effect)**									
ARA	11(S)-HETE	11-LOX	WT/HFD - KO/HFD	0.942	0.244	0.002	2.368	1.426	2.715
	15(S)-HETE	15-LOX		0.417	0.143	0.021	2.436	2.019	1.512
	PGE2–PGD2–PGJ2	COX		0.783	0.217	0.004	4.926	4.143	2.192
	6-keto PGF1a	COX + non-enzymatic		0.651	0.196	0.008	1.452	0.801	1.961
	8-iso PGF2a	ROS		0.569	0.221	0.044	2.844	2.274	1.764
	15-deoxy-delta12,14-PGJ2	Hydrolases		0.499	0.132	0.004	−0.143	−0.642	1.674
DHA	Resolvin D1–Resolvin D3–Resolvin D2	12/15-LOX		−0.224	0.062	0.008	0.451	0.675	0.797
**WT on STD vs. HFD (diet effect)**									
ARA	11(S)-HETE	11-LOX	WT/STD - WT/HFD	−1.322	0.255	0.000	1.045	2.368	0.243
	15(S)-HETE	15-LOX		−0.559	0.148	0.006	1.877	2.436	0.572
	PGE2–PGD2–PGJ2	COX		−1.021	0.225	0.001	3.905	4.926	0.354
	6-keto PGF1a	COX + non-enzymatic		−0.688	0.205	0.008	0.764	1.452	0.484
	8-iso PGF2a	ROS		−0.735	0.228	0.021	2.108	2.844	0.481
	15-deoxy-delta12,14-PGJ2	Hydrolases		−0.495	0.138	0.004	−0.638	−0.143	0.601
**WT vs. KO on STD (genotype effect)**									
ARA	PGE2–PGD2–PGJ2	COX	WT/STD - KO/STD	−0.424	0.174	0.030	3.905	4.329	0.647

*Brain oxylipin pathways are grouped according to main biosynthetic pathways by (1) FA precursors (i.e., LA, ARA, EPA, and DHA), oxylipin groups (i.e., midchain HODE, EET, mid-chain HETE, EpDPA, DiHDPA), (2) enzymes involved in their synthesis [i.e., oxygenation of PUFAs by LOX followed by reduction or alternatively hydroxylation of PUFAs by CYP1B1; oxidation of PUFAs by CYP450 followed by hydroxylation of oxidized PUFAs by soluble epoxide hydrolase (sEH)], and (3) based on enzymatic product to substrate ratio (i.e., hydroxylation of 10,11-EpDPA to 10,11-DiHDPA, 14,15-EET to 14,15-DiHET, or 19,20-EpDPA to 19,20-DiHDPA by sEH). AA, arachidonic acid; DHA, docosahexaenoic acid; LOX, lipoxygenase; PGD2, prostaglandin D2; PGE2, prostaglandin E2; PGJ2, prostaglandin J2; 11-HETE, 11-hydroxyeicosatrienoic; 15-HETE, 15-hydroxyeicosatrienoic acid; 20-HETE, 20-hydroxyeicosatrienoic acid; 14,15-DiHET, 14,15-dihydroxyeicosatrienoic acid. p-values: are the two-sided p-values associated with the observed statistic (based on t-test form fitting linear regression model), adjusted based on Benjamini–Hochberg false discovery rate correction method for multiple hypothesis testing. Estimate: is the estimated value of the regression term (estimate of coefficient of difference between two groups), SE: The standard error of the regression term (estimate of coefficient of difference between two groups), and fold change: ratio of one group to another group based on the marginal means.*

**TABLE 3:** Plasma oxylipins in GPR39 KO and WT littermate mice fed with standard diet and high fat diet.

Class	Oxylipins	Genotype/diet groups	Estimate	SE	*p*-Value	Mean first group	Mean second group	Fold change
**WT vs. KO on HFD (diet + genotype effect)**								
EPA	5,6-DiHETE	WT/HFD - KO/HFD	0.878	0.349	0.049	1.473	0.595	2.135
**WT on STD vs. HFD (diet effect)**								
ARA	20-HETE	WT/STD - WT/HFD	−2.016	0.570	0.009	−0.954	1.061	0.143
	PGD2		2.516	0.710	0.009	0.145	−2.370	12.905
DHA	7,8-DiHDPA		0.081	0.026	0.024	0.648	0.567	1.085
	10,11-DiHDPA		0.043	0.015	0.039	0.748	0.705	1.044
	7,8-EpDPA		0.073	0.022	0.018	0.195	0.122	1.076
	16,17-EpDPA		0.275	0.040	0.000	−0.995	−1.270	1.317
EPA	18-HEPE		2.470	0.334	0.000	3.960	1.490	11.271
	5,6-DiHETE		1.433	0.349	0.002	2.906	1.473	4.133
	8,9-DiHETE		1.702	0.364	0.001	−0.655	−2.357	4.906
	17,18-EpETE		2.715	0.288	0.000	0.527	−2.187	14.509
Linoleic acid	13(S)-HODE		1.654	0.425	0.004	2.781	1.127	5.301
	9,10-EpOME		1.537	0.529	0.040	0.620	−0.918	4.475
	9,10-DiHOME		0.815	0.239	0.012	2.131	1.317	2.287
α-Linolenic acid	9(S)-HOTrE		1.527	0.371	0.002	−0.781	−2.308	4.336

*Plasma oxylipins are grouped according to main biosynthetic pathways by (1) FA precursors (i.e., LA, ARA, EPA, and DHA), oxylipin groups (i.e., midchain HODE, EET, mid-chain HETE, EpDPA, DiHDPA), (2) enzymes involved in their synthesis [i.e., oxygenation of PUFAs by LOX followed by reduction or alternatively hydroxylation of PUFAs by CYP1B1; oxidation of PUFAs by CYP450 followed by hydroxylation of oxidized PUFAs by soluble epoxide hydrolase (sEH)], and (3) based on enzymatic product to substrate ratio (i.e., hydroxylation of 10,11-EpDPA to 10,11-DiHDPA, 14,15-EET to 14,15-DiHET, or 19,20-EpDPA to 19,20-DiHDPA by sEH). AA, arachidonic acid; DHA, docosahexaenoic acid; LOX, lipoxygenase; PGD2, prostaglandin D2; PGE2, prostaglandin E2; PGJ2, prostaglandin J2; 11-HETE, 11-hydroxyeicosatrienoic; 15-HETE, 15-hydroxyeicosatrienoic acid; 20-HETE, 20-hydroxyeicosatrienoic acid; 14,15-DiHET, 14,15-dihydroxyeicosatrienoic acid. p-values: are the two-sided p-values associated with the observed statistic (based on t-test form fitting linear regression model), adjusted based on Benjamini–Hochberg false discovery rate correction method for multiple hypothesis testing. Estimate: is the estimated value of the regression term (estimate of coefficient of difference between two groups), SE: The standard error of the regression term (estimate of coefficient of difference between two groups), and fold change: ratio of one group to another group based on the marginal means.*

**TABLE 4:** Plasma oxylipins pathways in GPR39 KO and WT littermate mice fed with standard diet and high fat diet.

Class	Oxylipins	Pathways	Genotype/diet groups	Estimate	SE	*p-*Value	Mean first group	Mean second group	Fold change
**WT vs. KO on HFD (diet + genotype effect)**									
ARA	20-HETE–11,12-EET–14,15-EET	CYPEPOX	WT/HFD - KO/HFD	0.057	0.023	0.035	2.930	2.873	1.058
**WT on STD vs. HFD (diet effect)**									
ARA	20-HETE–11,12-EET–14,15-EET	CYPEPOX	WT/STD - WT/HFD	−0.101	0.024	0.001	2.828	2.930	0.904
DHA	7,8-EpDPA–10,11-EpDPA–13,14-EpDPA	CYPEPOX		0.219	0.063	0.010	1.768	1.549	1.247
	17(R)-Resolvin D1–Resolvin D1–Resolvin D2	12/15-LOX		0.016	0.005	0.007	3.090	3.074	1.017
EPA	8-iso PGF3a–18-HEPE—NA	ROS		2.454	0.327	0.000	3.963	1.510	11.112
Linoleic acid	13(S)-HODE	12/15-LOX or ROS		1.654	0.425	0.004	2.781	1.127	5.301

*Plasma oxylipin pathways are grouped according to main biosynthetic pathways by (1) FA precursors (i.e., LA, ARA, EPA, and DHA), oxylipin groups (i.e., midchain HODE, EET, mid-chain HETE, EpDPA, DiHDPA), (2) enzymes involved in their synthesis [i.e., oxygenation of PUFAs by LOX followed by reduction or alternatively hydroxylation of PUFAs by CYP1B1; oxidation of PUFAs by CYP450 followed by hydroxylation of oxidized PUFAs by soluble epoxide hydrolase (sEH)], and (3) based on enzymatic product to substrate ratio (i.e., hydroxylation of 10,11-EpDPA to 10,11-DiHDPA, 14,15-EET to 14,15-DiHET, or 19,20-EpDPA to 19,20-DiHDPA by sEH). AA, arachidonic acid; DHA, docosahexaenoic acid; LOX, lipoxygenase; PGD2, prostaglandin D2; PGE2, prostaglandin E2; PGJ2, prostaglandin J2; 11-HETE, 11-hydroxyeicosatrienoic; 15-HETE, 15-hydroxyeicosatrienoic acid; 20-HETE, 20-hydroxyeicosatrienoic acid; 14,15-DiHET, 14,15-dihydroxyeicosatrienoic acid. p-values: are the two-sided p-values associated with the observed statistic (based on t-test form fitting linear regression model), adjusted based on Benjamini–Hochberg false discovery rate correction method for multiple hypothesis testing. Estimate: is the estimated value of the regression term (estimate of coefficient of difference between two groups), SE: The standard error of the regression term (estimate of coefficient of difference between two groups), and fold change: ratio of one group to another group base.*

## Results

### GPR39 Deletion Reduces Plasma Insulin Without Affecting Weight Gain or Glucose Concentration

High-fat diet caused an increase in body weight in both genotypes, resulting in significant weight gains compared to mice on STD diet at the end of the study (WT: HFD 56.20 ± 1.39 vs. STD 37.25 ± 1.93; KO: HFD 56.00 ± 0.45 vs. STD 35.80 ± 1.24; Figure 1B). No differences were observed in starting weights or weight gain on HFD between WT and KO mice. Fasting blood glucose was significantly higher in mice on HFD vs. STD in both genotypes, with no differences between the two genotypes (Figure 1C). However, plasma insulin levels were significantly reduced in KO mice regardless of diet (Figure 1D). The latter finding is consistent with previous reports indicating that GPR39 is required for the increase insulin secretion under conditions of increased demand, such as age-dependent and diet-induced insulin resistance (Tremblay et al., 2009). The discrepancy between the changes in glucose and insulin in GPR39 KO mice has previously been observed by others (Tremblay et al., 2009). GPR39 augments insulin secretion under conditions of increased demand; e.g., age-dependent and diet-induced insulin resistance. The effect is in part related to the role of GPR39 in pancreatic cell hyperplasia associated with the development of insulin resistance. The contribution of GPR39 to insulin secretion and plasma glucose regulation is revealed under conditions of high demand and reduced β-cell function, where the increase in insulin resulting from GPR39 deletion would be matched by a corresponding rise in glucose.

### GPR39 Deletion Does Not Affect Measures of Anxiety on Either Diet

When activity levels were assessed in the open areas of the elevated zero maze, there was no effect of HFD in any genotype. However, HFD reduced activity in the closed areas of the elevated zero maze in Het mice (Supplementary Figure 1A). This effect was not seen in WT or KO mice. In contrast to activity levels, there was no effect of HFD on measures of anxiety in the elevated zero maze in any genotype. Consistent with the elevated zero maze data, there was a trend for a reduction in activity levels in the open field in Het mice on a HFD (Supplementary Figure 1B), with no effects on activity levels seen in WT or KO mice. Finally, there was a trend for a decrease in Het mice for time spent in the anxiety-provoking center of the open field (Supplementary Figure 1C) and the number of entries into the center (Supplementary Figure 1D).

### GPR39 Deficiency Impairs Memory Retention Regardless of Diet

**FIGURE 2:**
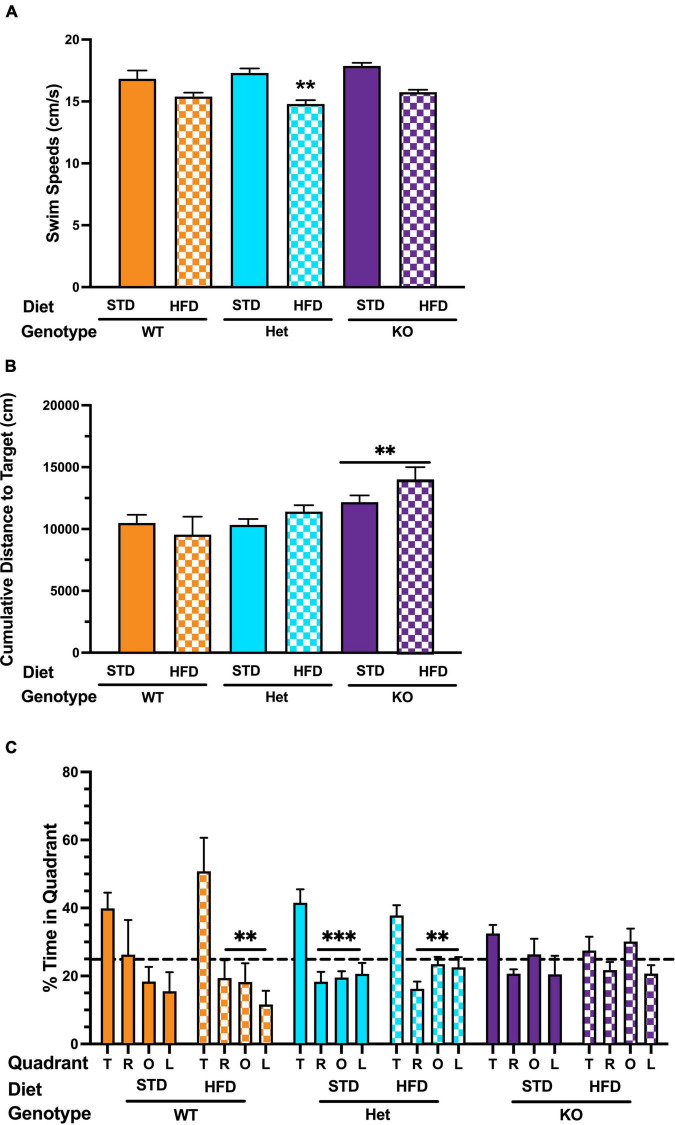
Effects of GPR39 deficiency on memory retention in the water maze. **(A)** Swim speeds during the visible platform showed an effect of diet in Het mice, with lower swim speeds in Het mice fed with HFD. **(B)** There was no effect of diet on latency or cumulative distance to the target in any genotype during visible or hidden platform training. However, KO mice fed with HFD took longer to locate the platform than KO mice fed with STD. **(C)** Spatial memory retention in the water maze probe trial showed an effect of quadrant in WT mice on a STD. However, they did not spend more time in the target quadrant than any non-target quadrant. WT on HFD spent more time in the target quadrant than any other quadrant. Het mice on a STD and HFD also spent more time in the target quadrant than any other quadrant. In contrast, no effect of quadrant was seen in KO mice on STD or HFD. All values represent means ± standard error of the mean, ^**^p < 0.001, ^***^p < 0.0001, *n* = 5–10 per group.

The time spent exploring the novel and familiar object in NOR was analyzed. There was a trend for KO mice on a HFD to spend more time exploring the novel object, but the difference did not reach statistical significance (*p* = 0.06969, Supplementary Figure 2). In the visible platform training of the water maze test, swim speed was lower in Het mice on HFD (Figure 2A), with a similar trend in KO mice, but the decrease did not reach statistical significance (*p* = 0.071, Figure 2A). There was no effect of diet on latency or cumulative distance to target in any genotype during visible platform training (Supplementary Figure 3). During hidden platform training, cumulative distance to target was significantly higher in KO mice compared to WT or Het, regardless of diet (Figure 2B). Because of the effect of HFD on swim speeds, we also analyzed distance moved as a performance measure. HFD did not reduce the ability to locate the visible platform in any genotype. During the hidden platform training, there was a trend for HFD to reduce distance moved in WT mice that did not reach statistical significance (*p* = 0.081). Next, spatial memory retention was assessed in the water maze probe trial (no platform). In WT mice on STD there was an effect of quadrant (*p* = 0.092), with a trend for more time spent in the target quadrant that was not statistically significant (Figure 2C). Interestingly, a strong spatial bias was seen in WT on the HFD, with mice spending significantly more time in the target quadrant than any other quadrant (*p* = 0.003). Het mice on either STD or HFD also showed spatial bias and spent more time in the target quadrant than any other quadrant (Figure 2C). In contrast, no effect of quadrant was seen in KO mice regardless of diet; the mice spent a similar amount of time in all quadrants (Figure 2C). Finally, fear learning and memory were assessed. When activity levels were analyzed during the baseline period (prior to the first tone), there was an effect of diet in Het mice, with lower activity levels in Het mice on a HFD in fear conditioning (Supplementary Figure 4A). This effect was genotype-dependent and not seen in WT or KO mice. When motion during the shocks was analyzed, there was an effect of diet in WT mice, with lower responses to the shock in WT mice on a HFD (Supplementary Figure 4B). This was not seen in Het or KO mice. There was no effect of diet on freezing during the tones or the inter-stimulus intervals in any genotype. There was no effect of diet on freezing during the contextual or cued fear memory tests (Supplementary Figure 4C). Taken together, these results indicate that GPR39 deletion impairs spatial memory retention and suggest a protective role for GPR39 against cognitive impairment.

### GPR39 Deficiency Promotes Systemic Inflammation

**FIGURE 3:**
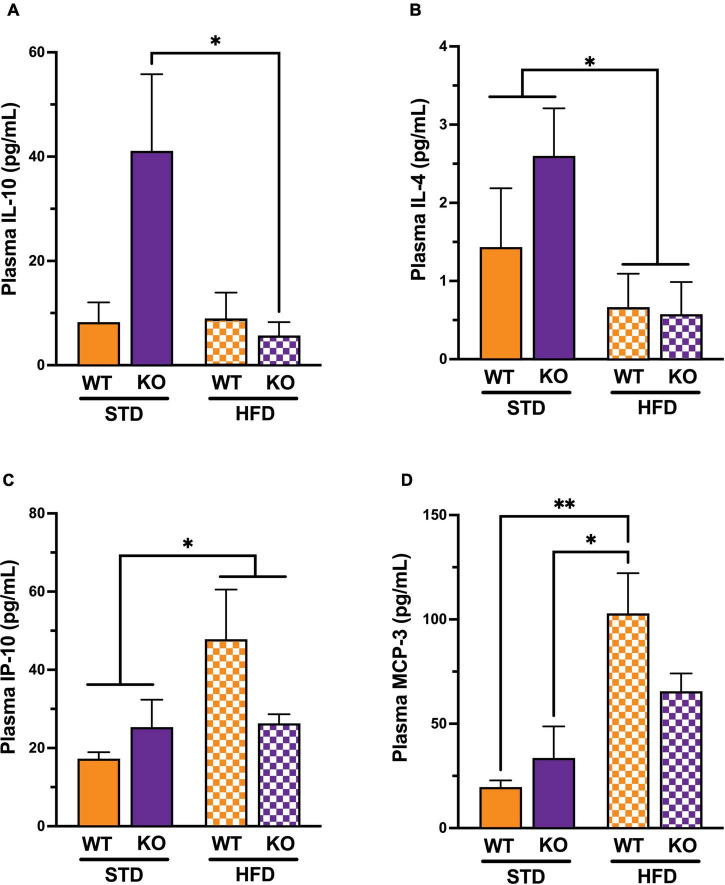
Effects of GPR39 deficiency on systemic inflammation. **(A)** IL-10 showed a significant interaction between genotype and diet and a significant diet effect. The *post-hoc* test revealed a significant difference between KO fed with HFD and KO fed with STD, whereas no significant difference existed between WT regardless of the diet. **(B)** IL-4 showed a significant diet effect between mice on STD compared to mice on HFD, whereas no significant difference existed between genotypes. **(C)** IP-10 showed a significant diet effect between mice on STD compared to mice on HFD, whereas no significant difference existed between genotypes. **(D)** MCP-3 showed a significant diet effect between mice on STD compared to mice on HFD, whereas no significant difference existed between genotypes. The *post-hoc* test revealed a significant difference between WT fed with HFD and WT fed with STD and a significant difference between WT fed with HFD and KO fed with STD without genotype effect. All values represent means ± standard error of the mean, *p < 0.05, ^**^p < 0.001, *n* = 3–4 per group.

Because there was no observed cognitive deficit in the Het group, we decided to remove it from the rest of the study. To characterize the differences in inflammatory state in mice, 26 protein markers of systemic inflammation were measured in plasma (Supplementary Table 1). Cytokine/chemokines multiplex analysis showed a significant interaction between genotype and diet and a significant diet effect (Figure 3A). The *post-hoc* analysis revealed a significant difference in IL-10 between KO mice on HFD vs. STD, with no such difference in WT. There was a statically significant difference in IL-4 between mice on STD vs. HFD (Figure 3B), with no statistically significant differences between WT and KO. Similarly, IP-10 was significantly higher in mice on HFD vs. STD (Figure 3C), with differences between WT and KO. Lastly, MCP-3 was higher in WT on HFD vs. STD, with no diet effect in the KO (Figure 3D). Regardless of diet, a trend for a decrease in IL-27 (*p* = 0.0523) in KO was observed without reaching statistical significance.

### Effect of GPR39 Deletion on Brain and Plasma Oxylipins

We next investigated whether GPR39 deficiency altered levels of specific oxylipins in brain and plasma. The data spread for oxylipins levels in the brain (in nanomoles, nM) is presented as the interquartile range (IQR) in Supplementary Tables 2, 3. Linear regression analysis was performed to compare experimental groups. Brain and plasma oxylipins were grouped according to the main biosynthetic pathways by (1) fatty acid (FA) precursors (i.e., LA, ARA, EPA, and DHA), oxylipin groups (i.e., midchain hydroxy-octadecadienoic acid (HODE), epoxy-eicosatrienoic acid (EET), mid-chain hydroxy-eicosatetraenoic acid (HETE), epoxy-docosapentaenoic acid (EpDPA), dihydroxy-docosapentaenoic acid (DiHDPA), (2) enzymes involved in their synthesis [i.e., oxygenation of PUFAs by LOX followed by reduction, or alternatively hydroxylation of PUFAs by CYP1B1; oxidation of PUFAs by CYP450 followed by hydroxylation of oxidized PUFAs by soluble epoxide hydrolase (sEH)], and (3) based on enzymatic product-to-substrate ratio (i.e., hydroxylation of 10,11-EpDPA to 10,11-DiHDPA, 14,15-EET to 14,15-DiHET, or 19,20-EpDPA to 19,20-DiHDPA by sEH) and reported in Tables 1, 2. Brain oxylipins analysis showed decreases in the concentrations of 11(S)-HETE, 15(S)-HETE, PGE2, PGD2, PGJ2, 6-keto PGF1a, 8-iso PGF2a, 11 b-PGF2a, 15-keto, PGF2a, 13,14-dihydro-15-keto PGE2, 13,14-dihydro-15-keto PGD2, 15-deoxy-delta12,14-PGJ2, thromboxane B2, 14,15-DiHET, and 7,8-DiHDPA in KO mice fed with HFD compared to WT mice fed with HFD. However, brain oxylipins showed increases in the concentrations of 11(S)-HETE, 15(S)-HETE, PGE2, PGD2, PGJ2, 6-keto PGF1a, 8-iso PGF2a, 11 b-PGF2a, 15-keto, PGF2a, 13,14-dihydro-15-keto PGE2, 13,14-dihydro-15-keto PGD2, 15-deoxy-delta12,14-PGJ2, Thromboxane B2, 14,15-DiHET, and 7,8-DiHDPA when comparing WT mice fed with HFD to WT mice fed with STD. While only PGE2, PGD2, 15-keto PGF2a, and Resolvin D1 appeared to be increased in KO mice fed with STD compared to WT mice fed with STD (Table 1). Fifteen oxylipins in the AA pathway identified above decrease significantly in response to HFD and GPR39 deletion, while only Resolvin D1 and 7,8-DiHDPA in the DHA pathway were identified to increase in response to HFD and GPR39 KO for individual oxylipins. Except Resolvin (D1, D2, and D3) in the 12/15-LOX pathway which increased in KO mice fed with HFD compared to WT mice fed with HFD, six oxylipins 11(S)-HETE, 15(S)-HETE, PGE2-PGD2-PGJ2, 6-keto PGF1a, 8-iso PGF2a, and 15-deoxy-delta12,14-PGJ2 in the AA pathway were identified to significantly decrease in response to HFD in KO mice compared to WT mice, while increasing in WT mice fed with HFD compared to WT mice fed with STD. In addition, prostaglandins (E_2_, D_2_ and J_2_) significantly increased in KO mice on STD compared to WT mice on STD (Table 2). The changes in the composition of these brain oxylipins were mainly associated with AA-derived oxylipins pathway. Like the brain, plasma oxylipins were also analyzed and results are presented in Tables 3, 4. Individual plasma oxylipins show a significant decrease in 5,6-DiHETE (EPA pathway) in KO mice on HFD compared to WT mice on HFD (Table 3). In addition, a significant effect of HFD was only seen in WT mice for 15 metabolites within five pathways: ARA, DHA, EPA, LA, and ALA (Table 3). Interestingly, HFD was associated with a significant increase in 20-HETE and a significant decrease in PGD_2_ in WT mice. Levels of 20-HETE-11,12-EET-14,15-EET (CYPEPOX pathway) were reduced in KO mice on HFD compared to WT mice on HFD (Table 4). The 20-HETE-11,12-EET-14,15-EET (CYPEPOX pathway) was increased by HFD in WT mice, while four metabolites in the ARA, DHA, EPA, and LA pathways were decreased by HFD only in WT mice (Table 4). Taken together, these results highlight changes in brain and plasma oxylipins by HFD and GPR39 gene deletion that are consistent with inflammation.

### GPR39 Deletion Reduces Resting Cerebral Blood Flow Without Affecting CO_2_-Induced Hypercapnia

**FIGURE 4:**
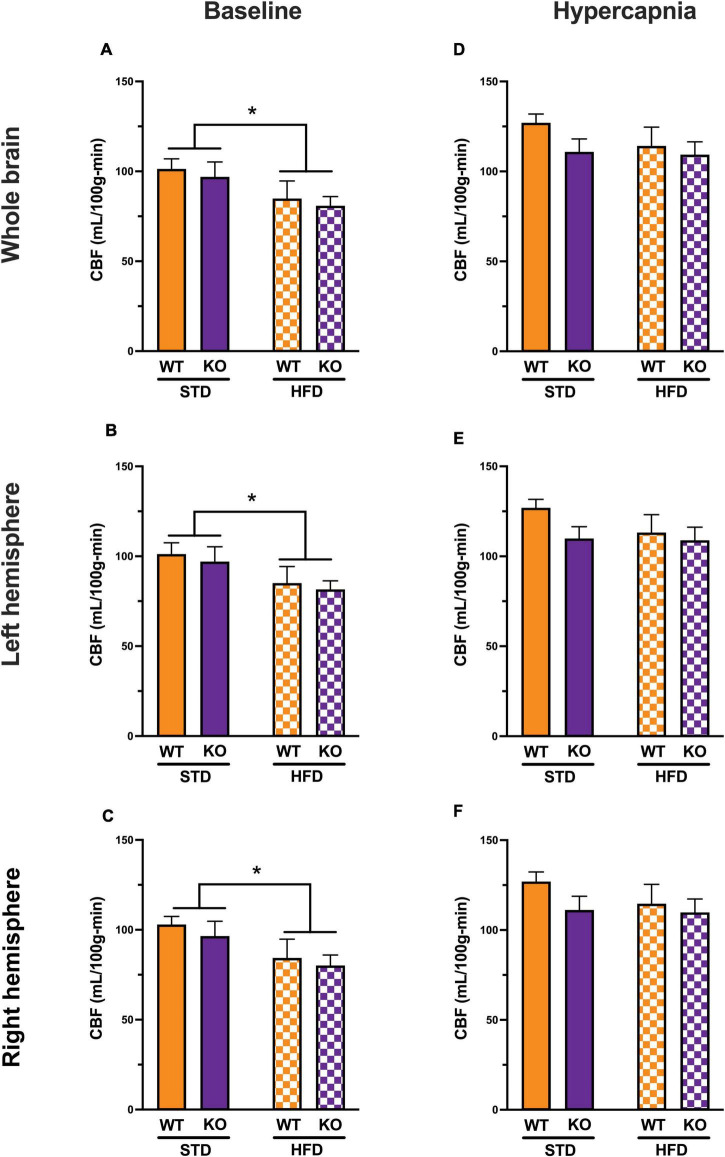
Effects of GPR39 deficiency on cerebral blood flow. **(A)** At baseline, CBF (in ml/100 g/min) showed a diet effect between STD and HFD mice with lower CBF in HFD, but without genotype effect for the whole brain. **(B,C)** Left and right hemispheres showed a diet effect between STD and HFD mice with lower CBF in HFD, but without genotype effect. **(D–F)** With hypercapnia (CO_2_ challenge), the absolute mean CBF increased in all groups with no diet or genotype effect for the whole brain and for both brain hemispheres. All values represent means ± standard error of the mean, *p < 0.05, *n* = 4–7 per group.

**FIGURE 5:**
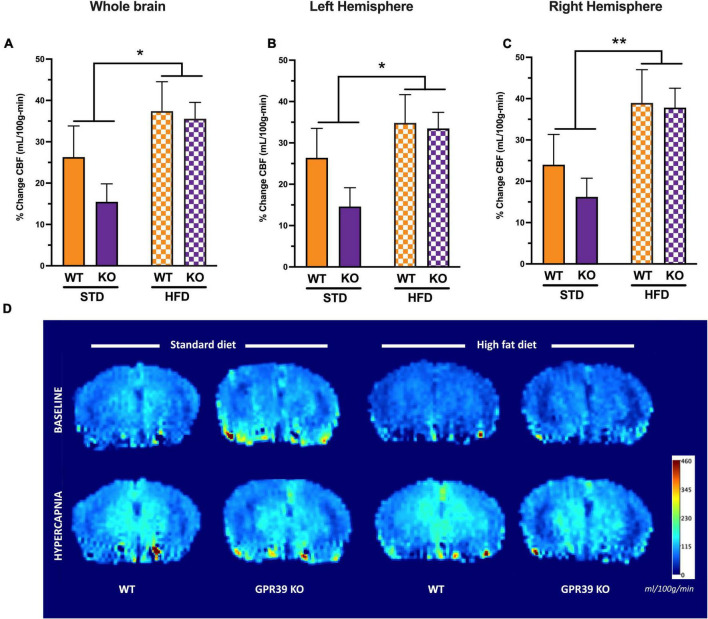
Effects of GPR39 deficiency on cerebrovascular reactivity. **(A–C)** Cerebrovascular reactivity to CO_2_ challenge showed a diet effect without genotype effect for the whole brain and for both brain hemispheres with a trend toward higher CBF in HFD fed mice compared to STD mice. **(D)** Grouped color perfusion maps of cerebral blood flow (mL/100g/min) showing reduced CBF associated with HFD. All values represent means ± standard error of the mean, *p < 0.05, **p < 0.001, *n* = 4–7 per group.

At baseline, HFD reduced CBF in the entire brain (Figure 4A) with no difference between WT and KO. The effect of HFD persisted when CBF was analyzed for each hemisphere separately; again, without difference between WT and KO (Figures 4B,C). With hypercapnia (CO_2_ challenge), the absolute mean CBF increased in all groups with no effect of HFD diet or GPR39 deletion for the whole brain CBF (Figure 4D) and for CBF in the left and right hemispheres (Figures 4E,F). When whole brain CBF was expressed as a percent change from baseline in response to hypercapnia [reflecting cerebrovascular reactivity (CVR) to CO_2_ challenge], we observed higher CVR in HFD vs. STD in both genotypes, with no differences between WT and KO mice (Figure 5A). Similarly, when evaluated separately for left and right brain hemisphere, the augmenting effect of HFD but not genotype on CVR was still observed (Figures 5B,C). Overall, these results indicate that HFD is associated with a significant reduction in resting CBF, but paradoxically, a significant increase in the ability to dilate, likely reflecting a larger difference between the lower baseline and maximum dilation (Figure 5D).

## Discussion

The primary goal of our study was to determine if GPR39 plays a role in cognitive impairment related to HFD. Based on the genetic association of GPR39 SNPs with markers of VCI and changes in GPR39 expression in postmortem human brain, we tested the hypothesis that GPR39 plays a protective role against cognitive impairment, mediated in part via oxylipins actions on CBF and neuroinflammation. We report that GPR39 gene deletion in KO mice causes spatial memory deficit, associated with changes in oxylipins and inflammatory mediators, with no effects on CBF.

GPR39 has been proposed to play a role in multiple homeostatic processes in the brain, including the balance between neuronal excitation and inhibition, vasodilation and vasoconstriction, and pro- and anti-inflammatory pathways (Xu et al., 2021). GPR39 has also been implicated in a variety of neurological and neuropsychiatric disorders, including AD, depression, anxiety, and seizures (Laitakari et al., 2021). Finally, GPR39 has been implicated in vascular pathology, including vascular inflammation and calcification. These diverse roles are believed to be related to the function of GPR39 as a receptor for zinc. More recently, we found that GPR39 also serves as a dual sensor for two eicosanoids with opposing actions on microvascular tone: the vasodilator 14,15-epoxyeicosatrienoate (14,15-EET) and vasoconstrictor 15-hydroxyeicosatetraenoate (15-HETE) (Alkayed et al., 2022).

We have previously linked a deficiency in 14,15-EET to cognitive impairment due to vascular causes (Nelson et al., 2014). We have also reported that GPR39, the target for 14,15-EET, is primarily expressed in the human brain in pericapillary pericytes, which play an important role in control of capillary blood flow and in maintaining blood–brain barrier (BBB) integrity. GPR39 expression was also observed in microglia in human brains. Interestingly, GPR39 expression in pericytes is increased in aged human brains, and microglial expression is increased in aged human brains with VCI. We additionally linked homozygous GPR39 SNPs with higher white matter hyperintensity volume, further supporting a role for GPR39 in VCI (Davis et al., 2021a).

In the current study, we used GPR39 mutant mice, with homozygous (KO) and heterozygous (Het) deletion of GPR39, to test the hypothesis that it plays a protective role against cognitive impairment in the HFD mouse model of metabolic syndrome. We have previously reported that GPR39 is expressed in microglia in postmortem human brain with small vessel disease (SVD) and a history of dementia, whereas in normal healthy young brain, GPR39 is predominantly expressed in pericytes (Davis et al., 2021a), suggesting that GPR39 upregulation in microglia may play a compensatory role in SVD-related dementia. We chose the HFD model because GPR39 has previously been implicated in food intake, weight control, and insulin secretion. Multiple studies have linked GPR39 with energy metabolism including two studies of GPR39 KO mice, with one where body weight and food intake were reported to be normal (Tremblay et al., 2007), while the other noted higher body weight and fat composition with no change in food intake (Moechars et al., 2006). A report has also linked GPR39 loss with impaired insulin secretion (Holst et al., 2009), yet synthetic agonists of GPR39 fail to drive insulin secretion (Fjellstrom et al., 2015). GPR39 KO mice have not been reported to have a diabetic phenotype and appear to have normal insulin secretion under baseline conditions (Tremblay et al., 2007, 2009). However, studies conducted on older GPR39 KO mice fed with a high sucrose or high fat diet demonstrated higher glucose and decreased insulin secretion compared to their wild type littermates (Holst et al., 2009; Tremblay et al., 2009; Verhulst et al., 2011). Our study did not find differences in body weight or plasma glucose between WT and KO mice, but we confirm the observation by Holst et al. (2009) indicating that GPR39 KO mice have reduced capacity to produce insulin.

The main finding of our study is that GPR39 gene deletion impairs spatial memory retention in the Morris water maze test. In contrast to spatial memory, GPR39 KO mice did not have a deficit in object recognition or fear learning and memory, suggesting that GPR39 plays a specific role in maintaining spatial memory. The deficit in spatial memory was seen whether mice were on STD or HFD, suggesting that GPR39 plays a role in maintenance of normal spatial memory and protects against HFD-induced spatial memory deficit. One caveat to this conclusion is that HFD did not impair spatial memory in WT mice, which is likely related to the genetic background of these mice (C57Black/6N), which is related to, but not identical to C57Black/6J. Although C57BL/6N mice are derived from C57BL/6J, they exhibit numerous phenotypic differences compared to C57BL/6J, including differences in performance in the open field, light/dark transition, grip-strength, rotarod, acoustic startle and pre-pulse inhibition, and the Morris water maze tests (Simon et al., 2013). In contrast to the study by Mlyniec et al. (2014), who reported that GPR39 knockout mice exhibit depressive and anxiety-like phenotypes (Mlyniec et al., 2014) in the forced swim and tail suspension tests, GPR39 KO mice in our study did not exhibit anxiety-like behavior in the elevated zero maze or open field tests.

Mechanistically, GPR39 deletion may lead to cognitive impairment through vascular or inflammatory mechanisms. However, our data does not support a role for reduced CBF in cognitive impairment related to GPR39 deficiency. Consistent with our previous studies showing a link between microvascular perfusion and cognitive performance in a mouse model of HFD diet-induced obesity (Johnson et al., 2016, 2019), here we show a similar link between reduced cerebral blood flow with high fat diet regimen. Our results support a role for inflammatory processes as potential underlying mechanisms for cognitive impairment in GR39 KO mice. Plasma cytokines/chemokines analysis show a trend for an increase in anti-inflammatory cytokines IL-10 and IL-4 in GPR39 KO mice, likely as a compensatory response to GPR39 deletion. HFD causes systemic inflammation and suppresses IL-10 and IL-4, including the increase in IL-10 and IL-4 caused by GPR39 deletion. IP-10 plays a role in T cell activation and trafficking, and MCP-3 (CCL7) is a chemoattractant for several leukocytes, including T cells. Therefore, the increase in IP-10 and MCP-3 by HFD diet is consistent with neuroinflammation and leukocyte infiltration. There are trends for higher IP-10 and MCP-3 in GPR39 KO on STD, but the trends are for lower levels when mice are on HFD.

It is not known how GPR39 deletion modulates inflammatory mechanisms. One possibility is that GPR39 on immune cells directly regulates cytokines and chemokines expression. Another possibility is that GPR39, which is regulated by oxylipins in turn regulates oxylipins-synthetic enzymes as a negative feedback loop. In support of this idea, we report here that GPR39 deletion induces AA COX metabolites PGE_2_ and PGD_2_ and 15-keto PGF2a and DHA 12/15-LOX metabolite Resolvin D1, suggesting that constitutive GPR39 activity suppresses COX (likely COX-2) and 12/15-LOX. We also report that HFD induces oxidative stress and inflammation in the central nervous system, as evidenced by induction of COX (likely COX2) and downstream enzymes (e.g., PG synthases), LOX enzymes (11-LOX and 12/15-LOX) and hydrolases. Upregulation of hydrolases confirms our previous observation of increased soluble epoxide hydrolase activity and expression in postmortem human brain with a history of dementia and evidence of small vessel disease (Nelson et al., 2014). Dysregulation of oxylipins metabolism has been implicated in both Alzheimer’s disease and type 2 diabetes (Morris et al., 2019), suggesting that oxylipin mechanism in GPR39 KO mice might be shared with VCI pathophysiology.

The change in oxylipins in brains likely has important pathophysiological consequences. For example, PGD_2_ is converted to PGJ_2_ which in turn is converted to 15-deoxy-D^12,14^-prostaglandin J2 (15dPG J2) reported to induce apoptosis in human astrocytes and cortical neurons (Chattopadhyay et al., 2000; Rohn et al., 2001). PGD_2_ is also a naturally occurring eicosanoid produced in the brain by the meninges and choroid plexus (Urade and Hayaishi, 2011). Its production is increased in response to inflammatory stimuli (Saper et al., 2012). At physiological concentrations from 1 nM to 1 μM, PGD_2_ it promotes neuroprotection *via* DP1 in a cAMP-dependent manner (Liang et al., 2005) while high concentrations >1 μM, PGD_2_ itself causes apoptosis (Yagami et al., 2003). Studies in both humans (Wellen and Hotamisligil, 2003) and in mouse models (Coelho et al., 2013; Gomez-Hernandez et al., 2016) have shown that obesity is linked to increased secretion of plasma fatty acids from adipose tissue into the vascular system, which causes inflammatory responses in the periphery as well as in the central nervous system (Silva Figueiredo et al., 2017). This systemic inflammation could drive activity by circulating immune and inflammatory cells that may contribute to neurological dysfunction (Bracko et al., 2020). HFD fed mice show increased invasion of monocytes into the central nervous system compared to animals fed with standard diet (Buckman et al., 2014). Thus, HFD might increase the severity of vascular inflammation in the brain and exacerbate the capillary stalling and CBF deficits seen in HFD fed mice (Bracko et al., 2020). Moreover, synaptic pruning by activated microglia was found to be elevated in obese rodents (Bocarsly et al., 2015; Hao et al., 2016; Duffy et al., 2019) and such rewiring of neural connections could also contribute to HFD-associated cognitive impairment. On the other hand, Resolvin D1 is anti-inflammatory and its induction by GPR39 is likely a compensatory response to protect against neuroinflammation and cognitive impairment (Mostafa and Satti, 2020).

Our study has some limitations. We were not able to evaluate neuroinflammation, because the brains were used for oxylipins analysis. However, the profile of oxylipins increased by HFD and GPR39 deletion is suggestive of increased neuroinflammation. We recently reported that GPR39 is an oxylipins receptor (Alkayed et al., 2022); therefore, we report here valuable and unique oxylipins data that point to specific pathways altered in brain and plasma by HFD and GPR39 deletion, as potential mediators of cognitive impairment in these models. Another limitation is that we used a global GPR39 KO mouse model. There is currently no mouse model with a floxed GPR39 that can be used to investigate the effects of cell- and tissue-specific deletion of GPR39. Finally, while mechanistic inquiries are beyond the scope of the current study, the data presented here is the first description of cognitive impairment in GPR39 KO mice. As discussed in our review article (Xu et al., 2021), specific cellular mediators are being actively investigated and will be pursued in future studies.

In conclusion, GPR39 deletion is associated with a spatial memory deficits and changes in brain and plasma oxylipins. We also showed that GPR39 deletion promotes systemic inflammation, but does not affect cerebral blood flow and CVR. We have identified specific oxylipins and their synthetic enzymes as potential mediators of cognitive impairment in VCI. GPR39, inflammation, and oxylipins may serve as biomarkers and therapeutic targets in VCI.

## Data Availability Statement

The original contributions presented in the study are included in the article/Supplementary Material, further inquiries can be directed to the corresponding author.

## Ethics Statement

The animal study was reviewed and approved by Department of Comparative Medicine Institutional Animal Care and Use Committee at Oregon Health and Science University, Portland, OR, United States.

## Author Contributions

TB, CD, EA, and NA conceived and designed the study. TB, CD, EA, RP, MP, MB, MG-J, AM, and JC conducted the experiments. TB, RP, and YZ analyzed collected data. TB, CD, EA, YZ, GB, CM, JR, MP, MB, MG-J, AM, JC, and NA discussed and interpreted the data. TB and NA wrote the first draft of the manuscript. All co-authors provided feedback on the draft, made editorial changes, and contributed to the final version of the manuscript.

## Conflict of Interest

NA is co-inventor of technologies related to GPR39 that have been licensed by OHSU to Vasocardea. This potential conflict of interest has been reviewed and managed by OHSU. The remaining authors declare that the research was conducted in the absence of any commercial or financial relationships that could be construed as a potential conflict of interest.

## Publisher’s Note

All claims expressed in this article are solely those of the authors and do not necessarily represent those of their affiliated organizations, or those of the publisher, the editors and the reviewers. Any product that may be evaluated in this article, or claim that may be made by its manufacturer, is not guaranteed or endorsed by the publisher.

## Abbreviations

ASL: arterial spin labeling
AA: arachidonic acid
AD: Alzheimer’s disease
ALA: α-linolenic acid
CBF: cerebral blood flow
COX: cyclooxygenases
DHA: docosahexaenoic acid
EPA: eicosapentaenoic acid
GPR39: G-protein coupled receptor 39
HFD: high-fat diet
KO: knock-out
LA: linoleic acid
LOX: lipoxygenases
MRI: magnetic resonance imaging
MWM: Morris water maze
NOR: novel object recognition
VCI: vascular cognitive impairment
WT: wild-type
ZT: zeitgeber time.
