# Supplementary material for: GPR39 Deficiency Impairs Memory and Alters Oxylipins and Inflammatory Cytokines Without Affecting Cerebral Blood Flow in a High-Fat Diet Mouse Model of Cognitive Impairment
Source: Front Cell Neurosci. 2022 Jul 6;16:893030. doi: 10.3389/fncel.2022.893030 (PMC9298837; doi:10.3389/fncel.2022.893030)
Supplement: Supplementary file 1 [file Data_Sheet_1.pdf]

1 Supplementary Material

1.1.1 Elevated zero maze

Measures of anxiety were assessed in the elevated zero maze. The enclosure consisted of four sections (6 cm wide), alternating between open and closed sections. Mice were placed into an open area of the maze and allowed to explore for 10 minutes. An automated photobeam detection method was used to track mouse movements: distance moved (cm), time spent in the open and closed areas as well as crossings between the open and closed areas.

1.1.2 Open field

Mice were placed into a square arena. The total open field was 16 × 16 inches. The center square was 8 × 8 inches. Mice were allowed to explore for 10 minutes. Behavioral performance was tracked and scored using an automated video system (Ethovision 14.0 XT, Noldus, Sterling VA). Exploratory behavior was analyzed using total distance moved (cm) as outcome measure. Time spent in the more anxiety-provoking center of the open field was analyzed as well. Fear Conditioning

In this task, mice learn to associate a conditioned stimulus (CS, e.g. the environmental context, or a discrete cue) with a mild foot shock (unconditioned stimulus, US). CS-US pairings are preceded by a short habituation period, during which a baseline measure of locomotor activity is analyzed. Contextual fear conditioning is considered to be hippocampus- and amygdala-dependent, while cued fear conditioning is considered to be hippocampus independent. Freezing, defined as immobility with the exception of respiration, is considered a post-exposure fear response, and is a widely used indicator of conditioned fear. Freezing depends on the threshold settings. Mice were trained and tested using a Med Associates mouse fear conditioning system containing Video Freeze automated scoring system (Med Associates, St. Albans, Vermont), as previously described in detail and validated against traditional hand scoring methods (51). On day 1, the mice were placed inside a dark fear-conditioning chamber. Chamber lights (at 100 lux) turned on at zero seconds, followed by a 90-second habituation period and a subsequent 30-second (2800 Hz, 80 dB) tone (cue). A 2-second 0.7 mA foot shock was administered at 28 seconds, co-terminating with the tone at 30 seconds. After a 30-second inter-stimulus-interval the tone-shock pairing were repeated for a total of five tone-shock pairings. On day 2, hippocampus dependent associative learning was assessed during re-exposure to the training environment for 300 seconds. Three hours later, mice were exposed to a modified environment (scented with vanilla extract, and cleaned with 10% isopropanol instead of 0.5% glacial acetic acid, novel floor texture covering the shock-grid, and rounded walls). They were allowed to habituate for 90 seconds, and then exposed to the cue for a second period of 180 seconds. Associative learning was measured as the percent time spent freezing in response to the contextual environment or the tone. Immediate acquisition of conditioned fear was measured following CS-US pairings. Motion during shock (proprietary

index, Med Associates) was measured to assess potential differences in response to the shock during training.

2 Supplemental figures and tables

Figure S1. Effects of GPR39 deficiency on anxiety

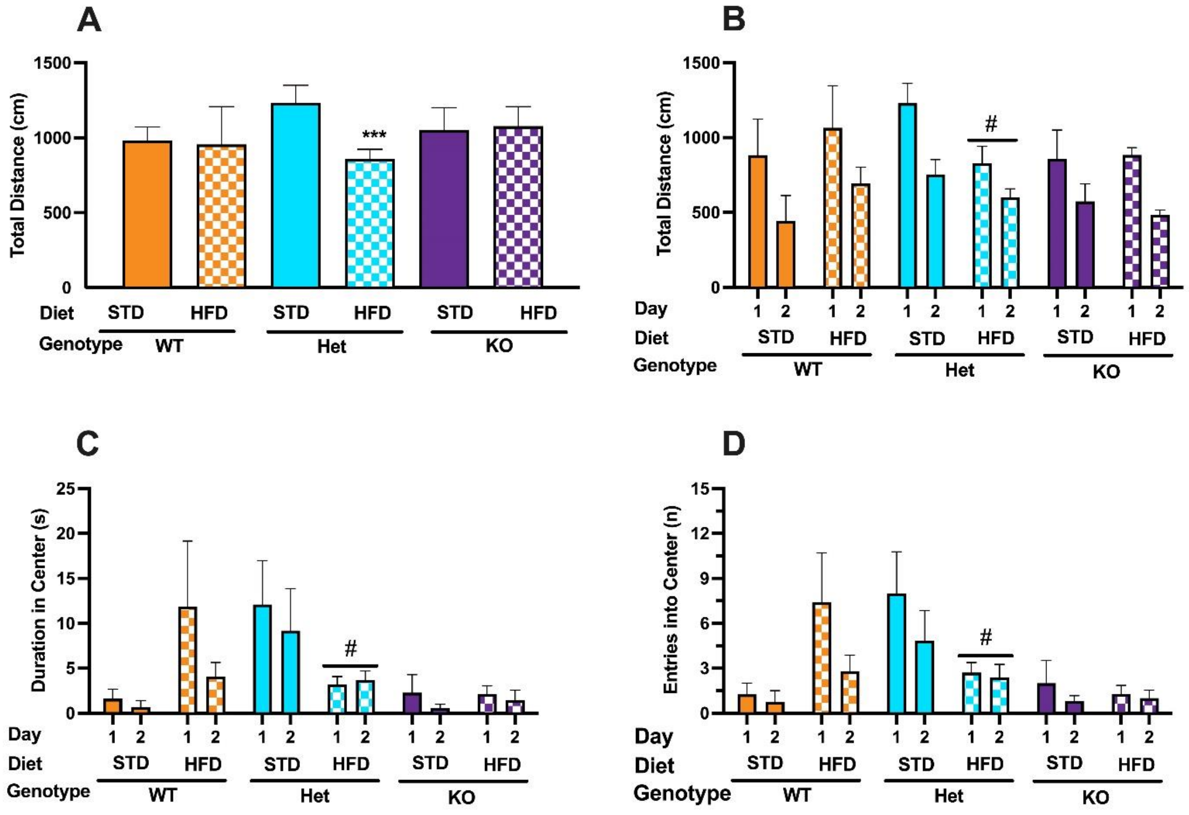

(**A**). Activity levels in the open areas of the elevated zero maze, showed no effect of diet in any genotype. However, there was an effect of diet on activity levels in the closed areas of the elevated zero maze in Het mice, with lower activity levels in Het mice on HFD. There was no effect of diet on measures of anxiety in the elevated zero maze in any genotype. (**B**). In the open field, there was a trend towards an effect of diet on activity levels, with a trend towards lower activity levels in HET mice on HFD. No effect or trend towards an effect on activity levels was seen in WT or KO mice. (**C**). Measures of anxiety in the more anxiety-provoking center of the open field showed a trend towards an effect of diet in Het mice for time spent in the center. (**D**). Number of entries into the center also showed a trend towards an effect of diet in Het mice, with lower entries in Het mice on HFD. All values represent means ± standard error of the mean, $***p < 0.0001$, $^{\#}p \geq 0.05$, n = 5-10 per group.

Figure S2. Effects of GPR39 deficiency on memory

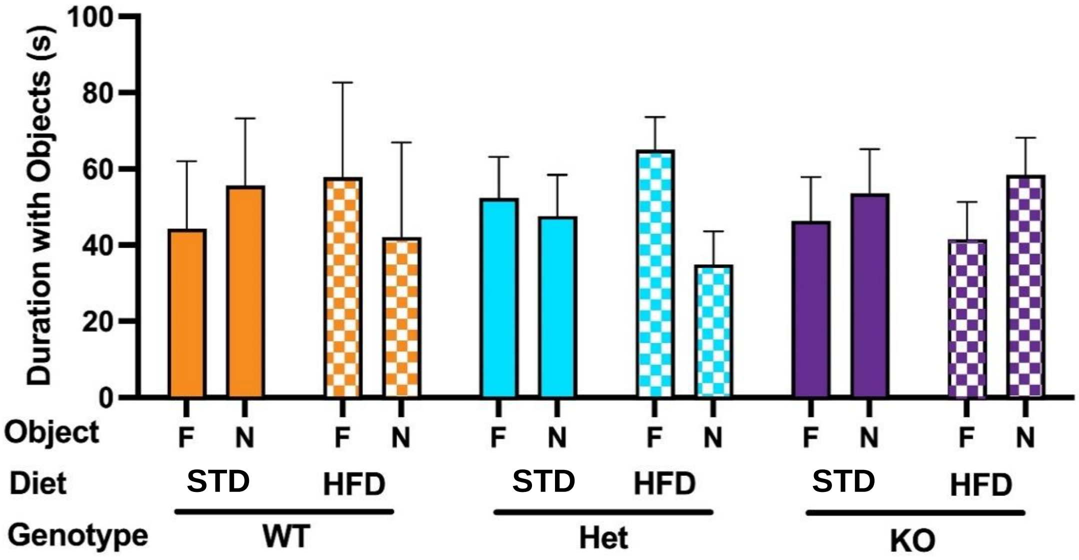

There was a trend towards KO mice on a HFD spending more time exploring the novel object, but it did not reach significance. All values represent means ± standard error of the mean, n = 5-10 per group.

Figure S3. GPR39 deficiency has no effect on latency or cumulative distance to target in any genotype

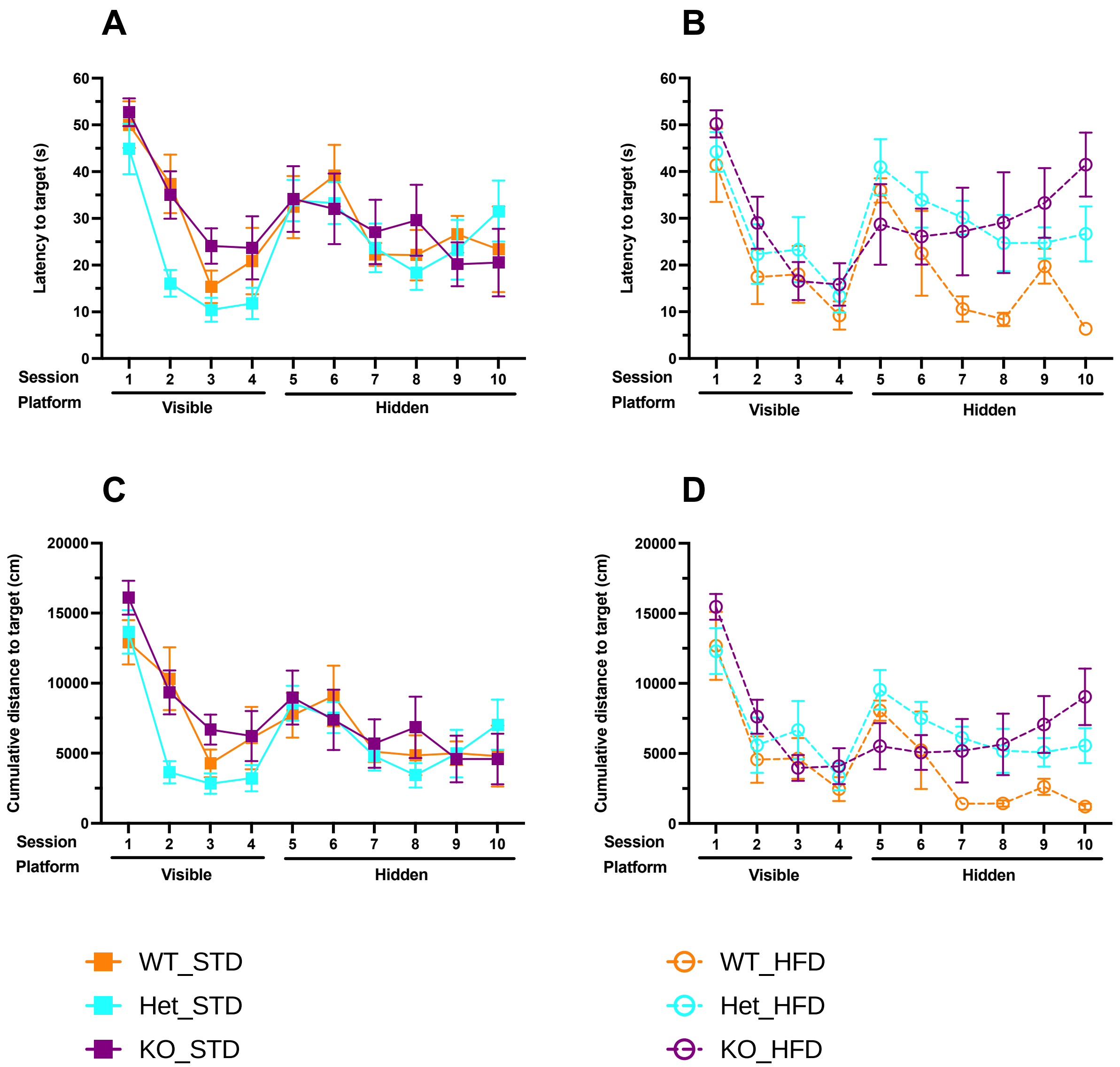

(**A-B**). There was no effect of diet on latency in any genotype during visible platform training. However, during the hidden platform training, there was a trend for KO mice fed with HFD to show an increased latency to the target compared to WT or Het fed with HFD, but that did not reach statistical significance. (**C-D**). Similar to latency to target, there was no effect of diet on cumulative distance to the target in any genotype during visible platform training. However, during the hidden platform training, there was a trend for KO mice fed with HFD to show an increased latency to target compared to WT or Het fed with HFD, but that did not reach statistical significance. All values represent means ± standard error of the mean, n = 5-10 mice per group.

Figure S4. Effects of GPR39 deficiency on fear learning

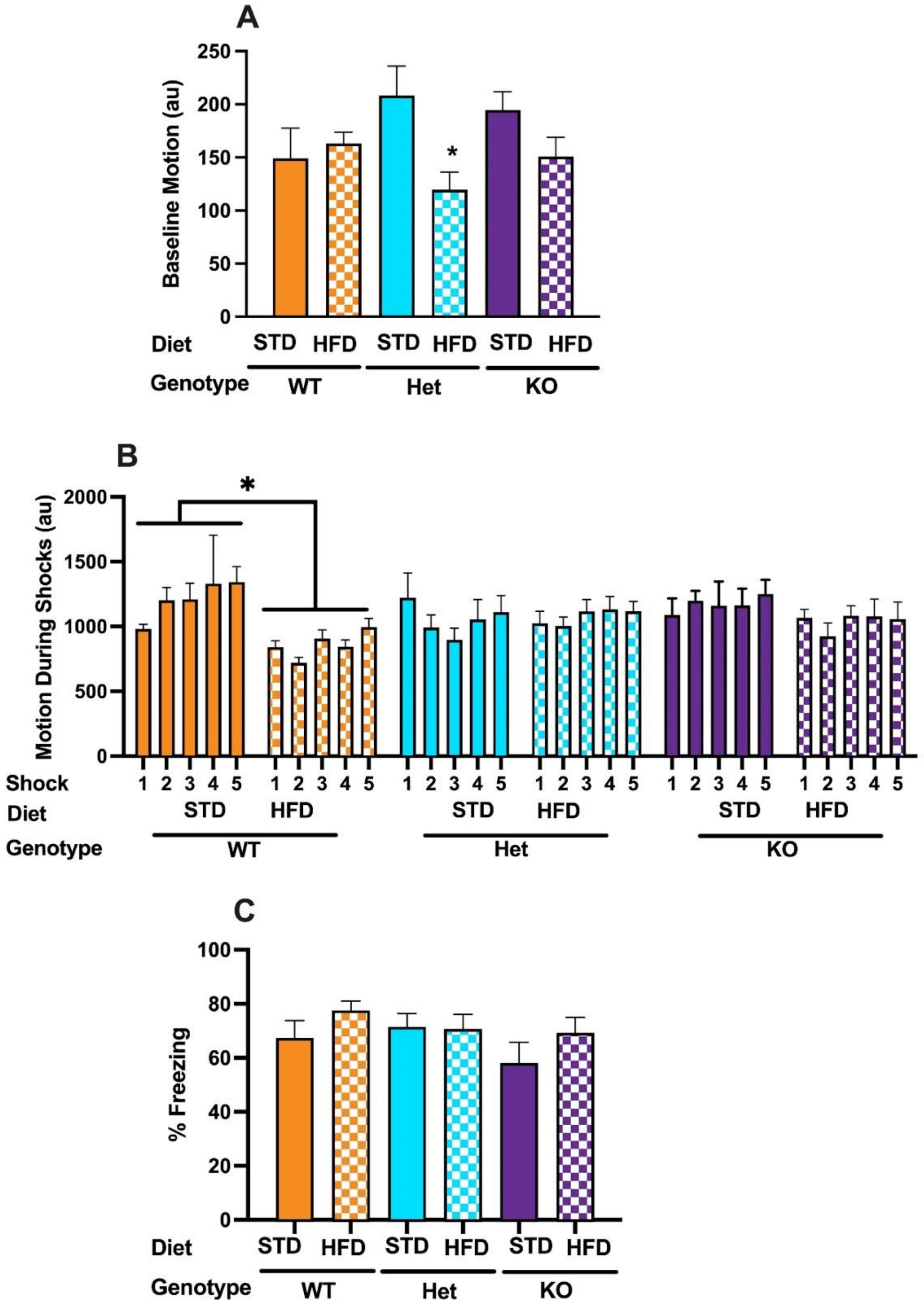

(**A**). During the shocks, motion showed an effect of diet in WT mice, with lower responses to the shock in WT mice on a HFD. This was not seen in Het or KO mice. (**B**). During the tones or the ISIs, there was no effect of diet on freezing in any genotype. (**C**). There was no effect of diet on freezing during the contextual or cued fear memory tests. All values represent means ± standard error of the mean, *$p < 0.05$, n = 5-10 per group.

Table S1: ProcartaPlex Mouse Cytokine & Chemokine Panel 1 26plex

Protein targets	Sensitivity	Standard Curve Range
**Th1/Th2**		
GM-CSF	0.19 pg/mL	2.4-10000 pg/mL
IFN gamma	0.09 pg/mL	1.2-5000 pg/mL
IL-1 beta	0.14. pg/mL	1.2-5000 pg/mL
IL-2	0.10 pg/mL	1.2-5000 pg/mL
IL-4	0.03 pg/mL	1.2-5000 pg/mL
IL-5	0.32 pg/mL	2.4-10000 pg/mL
IL-6	0.21 pg/mL	4.9-20000 pg/mL
IL-12p70	0.21 pg/mL	2.4-10000 pg/mL
IL-13	0.16 pg/mL	2.4-10000 pg/mL
IL-18	9.95 pg/mL	36.6-150000 pg/mL
TNF alpha	0.39 pg/mL	3.7-15000 pg/mL
**Th9/Th17/Th22/Treg**		
IL-9	0.28 pg/mL	18.3-75000 pg/mL
IL-10	0.69 pg/mL	2.4-10000 pg/mL
IL-17A (CTLA-8)	0.08 pg/mL	1.2-5000 pg/mL
IL-22	0.24 pg/mL	12.2-50000 pg/mL
IL-23	2.21 pg/mL	12.2-50000 pg/mL
IL-27	0.34 pg/mL	2.4-10000 pg/mL
**Chemokines**		
Eotaxin (CCL11)	0.01 pg/mL	0.5-2000 pg/mL
Gro alpha (CXCL1)	0.05 pg/mL	1.7-7000 pg/mL
IP-10 (CXCL10)	0.26 pg/mL	0.5-2000 pg/mL
MCP-1 (CCL2)	3.43 pg/mL	7.3-3000 pg/mL
MCP-3 (CCL7)	0.15 pg/mL	0.2-1000 pg/mL
MIP-1 alpha (CCL3)	0.13 pg/mL	0.5-2000 pg/mL
MIP-1 beta (CCL4)	1.16 pg/mL	1.2-5000 pg/mL
MIP-2	0.37 pg/mL	0.7-3000 pg/mL
RANTES (CCL5)	0.35 pg/mL	2.4-10000 pg/mL

Source: Cytokine & Chemokine 26-Plex Mouse ProcartaPlex™ Panel 1 (thermofisher.com)

Table S2. IQR brain oxylipins (nM) in GPR39 KO and WT littermate mice fed with standard diet and high fat diet.

Oxylipins (nM)	Pathway	WT STD	KO STD	WT HFD	KO HFD
		Median	Median	Median	Median
		(IQR)	(IQR)	(IQR)	(IQR)
**ARA**		4144.00	5503.66	4543.38	4153.23
		(3849.55, 4240.27)	(3774.30, 7225.03)	(4374.5, 7156.39)	(3247.80, 5367.27)
11(S)-HETE	11-LOX	2.92	3.90	10.00	4.18
		(2.26, 3.58)	(2.49, 4.74)	(6.12, 19.86)	(3.02, 5.66)
15(S)-HETE	15-LOX	6.45	7.69	12.08	7.63
		(6.09, 7.11)	(6.83, 8.30)	(10.80, 25.68)	(6.98, 8.99)
PGE2	COX	1.97	2.86	7.60	2.44
		(1.78, 2.09)	(2.46, 3.56)	(5.23, 18.69)	(2.03, 3.53)
PGD2		16.12	30.59	74.64	21.49
		(12.93, 18.33)	(22.32, 36.98)	(40.56, 121.91)	(16.40, 28.14)
PGJ2		1.71	2.15	4.40	1.82
		(1.55, 1.74)	(1.72, 2.91)	(2.85, 7.87)	(1.61, 2.36)
6-keto PGF1a	COX + non-enzymatic	2.17	2.41	4.31	2.17
		(2.00, 2.28)	(2.16, 2.69)	3.02, 6.00)	(2.01, 2.55)
8-iso PGF2a	ROS	8.34	10.40	18.67	10.25
		(7.32, 9.20)	(8.80, 11.79)	(15.81, 72.99)	(9.10, 12.05)
11 b-PGF2a	COX	8.89	11.50	22.01	9.93
		(8.15, 10.05)	(9.37, 11.86)	(17.52, 92.30)	(8.99, 13.54)
15-keto PGF2a		10.25	18.95	45.75	13.36
		(8.18, 11.42)	(13.77, 23.16)	(25.26, 75.04)	(10.37, 17.67)
13,14-dihydro-15-keto PGE2		0.66	0.68	0.73	0.66
		(0.64, 0.67)	(0.67, 0.69)	(0.73, 0.85)	(0.64, 0.69)
13,14-dihydro-15-keto PGD2		0.55	0.71	1.02	0.59
		(0.43, 0.69)	(0.62, 0.80)	(0.73, 1.50)	(0.51, 0.68)
15-deoxy-delta12,14-PGJ2	Hydrolases	0.51	0.64	0.86	0.53
		(0.47, 0.63)	(0.57, 0.68)	(0.66, 1.14)	(0.47, 0.62)
Thromboxane B2	COX	9.03	11.31	16.37	10.26
		(8.66, 9.30)	(9.70, 12.15)	(13.60, 36.18)	(9.42, 12.18)
14,15-DiHET	CYPEPOX/sEH	0.51	0.82	1.33	0.85
		(0.33, 0.67)	(0.72, 0.93)	(1.05, 3.39)	(0.48, 0.99)
**DHA**		2157.71	2701.07	2338.90	2197.25
		(1987.16, 2397.25)	(2268.72, 3456.37)	(2289.43, 4218.18)	(1790.83, 2511.75)
Resolvin D1	12/15-LOX	0.77	0.92	0.85	0.85
		(0.76, 0.89)	(0.87, 0.94)	(0.75, 0.85)	(0.79, 0.90)
7,8-DiHDPA	CYPEPOX/sEH	1.04	1.06	1.20	1.02
		(0.99, 1.08)	(1.02, 1.07)	(1.14, 1.33)	(0.99, 1.13)

Brain oxylipins are grouped according to main biosynthetic pathways by (1) FA precursors (i.e., LA, ARA, EPA, and DHA), oxylipin groups (i.e., midchain HODE, EET, mid-chain HETE, EpDPA, DiHDPA), (2) enzymes involved in their synthesis [i.e., oxygenation of PUFAs by LOX followed by reduction or alternatively hydroxylation of PUFAs by CYP1B1; oxidation of PUFAs by CYP450 followed by hydroxylation of oxidized PUFAs by soluble epoxide hydrolase (sEH)], and (3) based on enzymatic product to substrate ratio (i.e., hydroxylation of 10,11-EpDPA to

10,11-DiHDPA, 14,15-EET to 14,15-DiHET, or 19,20-EpDPA to 19,20-DiHDPA by sEH). AA indicates arachidonic acid; ADA, DHA, docosahexaenoic acid; LOX, lipoxygenase; PGD2: prostaglandin D2; PGE2: prostaglandin E2; PGJ2: prostaglandin J2; 11-HETE: 11-hydroxyeicosatrienoic; 15-HETE, 15-hydroxyeicosatrienoic acid; 20-HETE; 14,15-DiHET, 14,15-dihydroxyeicosatrienoic acid; dihydroxy-docosapentaenoic acid.

Table S3. IQR plasma oxylipins pathways in GPR39 KO and WT littermate mice fed with standard diet and high fat diet.

Oxylipins (nM)	Pathway	WT STD Median (IQR)	KO STD Median (IQR)	WT HFD Median (IQR)	KO HFD Median (IQR)
**ARA**		1032.41 (796.75, 1281.77)	897.77 (724.74, 1084.27)	973.54 (910.30, 1074.13)	979.29 (826.96, 1461.01)
20-HETE	CYPEPOX	0.35 (0.23, 0.74)	0.49 (0.20, 0.89)	2.53 (2.03, 4.71)	1.02 (0.50, 1.73)
PGD2	COX	1.47 (0.60, 2.03)	2.15 (1.84, 3.01)	0.07 (0.06, 0.18)	0.06 (0.02, 0.38)
**DHA**		1547.46 (1032.80, 2018.78)	1348.95 (1254.53, 1434.80)	731.06 (568.32, 745.94)	741.88 (598.86, 1396.09)
7,8-DiHDPA	CYPEPOX/sEH	1.88 (1.83, 2.03)	1.91 (1.85, 2.04)	1.77 (1.69, 1.83)	1.76 (1.73, 1.82)
10,11-DiHDPA	CYPEPOX/sEH	2.09 (2.08, 2.17)	2.10 (2.07, 2.17)	2.02 (2.00, 2.05)	2.01 (2.00, 2.03)
7,8-EpDPA	CYPEPOX	1.21 (1.18, 1.26)	1.23 (1.20, 1.28)	1.12 (1.11, 1.16)	1.12 (1.11, 1.15)
16,17-EpDPA	CYPEPOX	0.37 (0.36, 0.38)	0.37 (0.35, 0.47)	0.28 (0.28, 0.29)	0.30 (0.28, 0.33)
**EPA**		566.91 (468.38, 729.84)	597.25 (485.20, 642.74)	160.73 (80.91, 174.65)	131.42 (103.68, 277.42)
18-HEPE	ROS	52.56 (39.71, 71.19)	50.76 (32.89, 82.55)	5.50 (2.41, 6.59)	4.98 (4.06, 17.70)
5,6-DiHETE	CYPEPOX/sEH	17.53 (14.77, 23.93)	21.15 (11.11, 33.55)	3.99 (3.28, 6.33)	2.21 (0.68, 6.31)
8,9-DiHETE	CYPEPOX/sEH	0.52 (0.46, 0.59)	0.37 (0.27, 0.71)	0.10 (0.05, 0.17)	0.07 (0.03, 0.17)
17,18-EpETE	CYPEPOX/sEH	1.70 (1.58, 1.81)	2.11 (1.24, 2.94)	0.12 (0.08, 0.15)	0.13 (0.07, 0.54)
**Linoleic acid**		N/A	N/A	N/A	N/A
13(S)-HODE	12/15-LOX or ROS	16.43 (13.07, 19.83)	16.44 (12.44, 30.12)	2.90 (2.86, 3.55)	6.78 (1.86, 13.89)
9,10-EpOME	CYPEPOX	1.87 (1.32, 2.68)	1.75 (0.80, 4.39)	0.52 (0.21, 0.58)	0.67 (0.21, 1.78)
9,10-DiHOME	CYPEPOX/sEH	8.37 (6.40, 11.36)	8.86 (7.73, 17.15)	3.33 (3.15, 4.94)	3.40 (2.69, 5.56)
**a-Linoleic acid**		N/A	N/A	N/A	N/A
9(S)-HOTrE	LOX	0.47 (0.40, 0.51)	0.54 (0.26, 0.91)	0.13 (0.06, 0.13)	0.19 (0.09,).33)

Plasma oxylipins are grouped according to main biosynthetic pathways by (1) FA precursors (i.e., LA, ARA, EPA, and DHA), oxylipin groups (i.e., midchain HODE, EET, mid-chain HETE, EpDPA, DiHDPA), (2) enzymes involved in their synthesis [i.e., oxygenation of PUFAs by LOX followed by reduction or alternatively hydroxylation of PUFAs by CYP1B1; oxidation of PUFAs by CYP450 followed by hydroxylation of oxidized PUFAs by soluble epoxide hydrolase (sEH)], and (3) based on enzymatic product to substrate ratio (i.e., hydroxylation of 10,11-EpDPA to 10,11-DiHDPA, 14,15-EET to 14,15-DiHET, or 19,20-EpDPA to 19,20-DiHDPA by sEH). AA indicates arachidonic acid; ADA, DHA, docosahexaenoic acid; LOX, lipoxygenase; PGD2: prostaglandin D2; PGE2: prostaglandin E2; PGJ2: prostaglandin J2; 11-HETE: 11-hydroxyeicosatrienoic; 15-HETE, 15-hydroxyeicosatrienoic acid; 20-HETE; 14,15-DiHET, 14,15-dihydroxyeicosatrienoic acid; dihydroxy-docosapentaenoic acid.
